# Supplementary material for: A First Report of Sclerotinia sclerotiorum Causing Forsythia Twig Blight in Romania
Source: Plants (Basel). 2023 Oct 10;12(20):3516. doi: 10.3390/plants12203516 (PMC10610540; doi:10.3390/plants12203516)
Supplement: Supplementary file 1 [file plants-12-03516-s001.zip › plants-2632098-supplementary.pdf]

IDENTIFUNGO PLUS Report

Padova, 13/01/2023

Login:	amflorea
Booking:	176940
Sample name:	SS_F
Number of assembled sequences:	4
Database:	NCBI(nr)
Number of results:	10

The results of the IDENTIFUNGO PLUS service are as follows:

1) the consensus of the sanger sequences's assembly
2) the results of the similarity research obtained with the Blast software (http://blast.ncbi.nlm.nih.gov/Blast.cgi).

Consensus

```
>SS_F
CCCGAAGGGTAGACCTCCCACCCTTGTGTATTATTACTTTGTTGCTTTGGCGAGCTGCTCTTCGGGGCCTTGTATGCTCG
CCAGAGAATATCAAAACTCTTTTTATTAATGTCGTCTGAGTACTATATAATAGTTAAAACTTTCAACAACGGATCTCTTG
GTTCTGGCATCGATGAAGAACGCAGCGAAATGCGATAAGTAATGTGAATTGCAGAATTCAGTGAATCATCGAATCTTTGA
ACGCACATTGCGCCCCTTGGTATTCCGGGGGGCATGCCTGTTCGAGCGTCATTTCAACCCTCAAGCTCAGCTTGGTATTG
AGTCCATGTCAGTAATGGCAGGCTCTAAAATCAGTGGCGGCGCCGCTGGGTCCTGAACGTAGTAATATCTCTCGTTACAG
GTTCTCGGTGTGCTTCTGCCAAAACCCAAATTTTCTATGGTTGACCTCGGATCAGGTAGGGATACCCGCTGAACTTAAGC
ATATCAATAAGCGGAGGAAAAGAAACCAACAGGGATTACCTCAGTAACGGCGAGTGAAGCGGTAAAAGCTCAAATTTGAA
ATCTGGCTCTTTCAGAGTCCGAGTTGTAATTTGTAGAAGATGCTTCGGGTGTGGTTCCGGTCTAAGTTCCTTGGAACAGG
ACGTCATAGAGGGTGAGAATCCCGTATGTGACTGGATACCTATGCTCATGTGAAGCTCTTTCGACGAGTCGAGTTGTTTG
GGAATGCAGCTCAAAATGGGTGGTATATTTCATCTAAAGCTAAATATTGGCCAGAGACCGATAGCGCACAAGTAGAGTGA
TCGAAAGATGAAAAGCACTTTGGAAAGAGAGTTAAACAGTACGTGAAATTGTTGAAAGGGAAGCGCTTGCAATCAGACTT
GCACTTGGTGTTCATCAGGGTTTCGTGCCCTGTGTACTTCATCAAGTTCAGGCCAGCATCAGTTTGAGTGGTTAGATAAA
GGCTTGGAGAATGTGGCCCTCTTCGGGGGGTGTTATAGCTCCAGGTGCAATGTAGCCTACTTGGACTGAGGACCGCGCTT
CGGCTAGGATGCTGGCGTAATGGTTGTAAGCGAC
```

BLAST results

BLASTN 2.13.0+

Reference: Zheng Zhang, Scott Schwartz, Lukas Wagner, and Webb Miller (2000), "A greedy algorithm for aligning DNA sequences", J Comput Biol 2000; 7(1-2):203-14.

Reference for database indexing: Aleksandr Morgulis, George Coulouris, Yan Raytselis, Thomas L. Madden, Richa Agarwala, Alejandro A. Schaffer (2008), "Database Indexing for Production MegaBLAST Searches", Bioinformatics 24:1757-1764.

RID: W2S1AK1B013

```
Database: Nucleotide collection (nt)
           89,517,999 sequences; 981,602,536,810 total letters
Query= SS_F

Length=1074


                                                                  Score     E     Max
Sequences producing significant alignments:                      (Bits)  Value  Ident

CP017820.1 Sclerotinia sclerotiorum chromosome 7 sequence          1978    0.0    100%
KC311494.1 Sclerotinia sclerotiorum isolate KR1121_1 18S ribos...  1978    0.0    100%
MZ048353.1 Sclerotinia subarctica voucher WU 43983 small subun...  1925    0.0    100%
MZ048352.1 Stromatinia rapulum voucher WU 43986 small subunit ...  1905    0.0    99%
KR094468.1 Botrytis cinerea strain G409 18S ribosomal RNA gene...  1901    0.0    99%
CP009808.1 Botrytis cinerea B05.10 chromosome BCIN04, complete...  1901    0.0    99%
CP080982.1 Botrytis cinerea strain Sl3 chromosome 04               1901    0.0    99%
CP080964.1 Botrytis cinerea strain Vv3 chromosome 04               1901    0.0    99%
MW791983.1 Botrytis cinerea voucher culture Y224P internal tra...  1901    0.0    99%
MW791982.1 Botrytis cinerea voucher culture Y224O internal tra...  1901    0.0    99%

ALIGNMENTS
>CP017820.1 Sclerotinia sclerotiorum chromosome 7 sequence
Length=2434682

 Score = 1978 bits (1071),  Expect = 0.0
 Identities = 1074/1075 (99%), Gaps = 1/1075 (0%)
 Strand=Plus/Plus

Query  1        CCCG-AAGGGTAGACCTCCCACCCTTGTGTATTATTACTTTGTTGCTTTGGCGAGCTGCT  59
                |||| |||||||||||||||||||||||||||||||||||||||||||||||||||||||
Sbjct  2401598  CCCGAAAGGGTAGACCTCCCACCCTTGTGTATTATTACTTTGTTGCTTTGGCGAGCTGCT  2401657

Query  60       CTTCGGGGCCTTGTATGCTCGCCAGAGAATATCAAAACTCTTTTTATTAATGTCGTCTGA  119
                ||||||||||||||||||||||||||||||||||||||||||||||||||||||||||||
Sbjct  2401658  CTTCGGGGCCTTGTATGCTCGCCAGAGAATATCAAAACTCTTTTTATTAATGTCGTCTGA  2401717

Query  120      GTACTATATAATAGTTAAAACTTTCAACAACGGATCTCTTGGTTCTGGCATCGATGAAGA  179
                ||||||||||||||||||||||||||||||||||||||||||||||||||||||||||||
Sbjct  2401718  GTACTATATAATAGTTAAAACTTTCAACAACGGATCTCTTGGTTCTGGCATCGATGAAGA  2401777

Query  180      ACGCAGCGAAATGCGATAAGTAATGTGAATTGCAGAATTCAGTGAATCATCGAATCTTTG  239
                ||||||||||||||||||||||||||||||||||||||||||||||||||||||||||||
Sbjct  2401778  ACGCAGCGAAATGCGATAAGTAATGTGAATTGCAGAATTCAGTGAATCATCGAATCTTTG  2401837

Query  240      AACGCACATTGCGCCCCTTGGTATTCCGGGGGGCATGCCTGTTCGAGCGTCATTTCAACC  299
                ||||||||||||||||||||||||||||||||||||||||||||||||||||||||||||
Sbjct  2401838  AACGCACATTGCGCCCCTTGGTATTCCGGGGGGCATGCCTGTTCGAGCGTCATTTCAACC  2401897

Query  300      CTCAAGCTCAGCTTGGTATTGAGTCCATGTCAGTAATGGCAGGCTCTAAAATCAGTGGCG  359
                ||||||||||||||||||||||||||||||||||||||||||||||||||||||||||||
Sbjct  2401898  CTCAAGCTCAGCTTGGTATTGAGTCCATGTCAGTAATGGCAGGCTCTAAAATCAGTGGCG  2401957

Query  360      GCGCCGCTGGGTCCTGAACGTAGTAATATCTCTCGTTACAGGTTCTCGGTGTGCTTCTGC  419
                ||||||||||||||||||||||||||||||||||||||||||||||||||||||||||||
Sbjct  2401958  GCGCCGCTGGGTCCTGAACGTAGTAATATCTCTCGTTACAGGTTCTCGGTGTGCTTCTGC  2402017

Query  420      CAAAACCCAAATTTTCTATGGTTGACCTCGGATCAGGTAGGGATACCCGCTGAACTTAAG  479
                ||||||||||||||||||||||||||||||||||||||||||||||||||||||||||||
Sbjct  2402018  CAAAACCCAAATTTTCTATGGTTGACCTCGGATCAGGTAGGGATACCCGCTGAACTTAAG  2402077
```

```
Query  480      CATATCAATAAGCGGAGGAAAAGAAACCAACAGGGATTACCTCAGTAACGGCGAGTGAAG  539
                ||||||||||||||||||||||||||||||||||||||||||||||||||||||||||||
Sbjct  2402078  CATATCAATAAGCGGAGGAAAAGAAACCAACAGGGATTACCTCAGTAACGGCGAGTGAAG  2402137

Query  540      CGGTAAAAGCTCAAATTTGAAATCTGGCTCTTTCAGAGTCCGAGTTGTAATTTGTAGAAG  599
                ||||||||||||||||||||||||||||||||||||||||||||||||||||||||||||
Sbjct  2402138  CGGTAAAAGCTCAAATTTGAAATCTGGCTCTTTCAGAGTCCGAGTTGTAATTTGTAGAAG  2402197

Query  600      ATGCTTCGGGTGTGGTTCCGGTCTAAGTTCCTTGGAACAGGACGTCATAGAGGGTGAGAA  659
                ||||||||||||||||||||||||||||||||||||||||||||||||||||||||||||
Sbjct  2402198  ATGCTTCGGGTGTGGTTCCGGTCTAAGTTCCTTGGAACAGGACGTCATAGAGGGTGAGAA  2402257

Query  660      TCCCGTATGTGACTGGATACCTATGCTCATGTGAAGCTCTTTCGACGAGTCGAGTTGTTT  719
                ||||||||||||||||||||||||||||||||||||||||||||||||||||||||||||
Sbjct  2402258  TCCCGTATGTGACTGGATACCTATGCTCATGTGAAGCTCTTTCGACGAGTCGAGTTGTTT  2402317

Query  720      GGGAATGCAGCTCAAAATGGGTGGTATATTTCATCTAAAGCTAAATATTGGCCAGAGACC  779
                ||||||||||||||||||||||||||||||||||||||||||||||||||||||||||||
Sbjct  2402318  GGGAATGCAGCTCAAAATGGGTGGTATATTTCATCTAAAGCTAAATATTGGCCAGAGACC  2402377

Query  780      GATAGCGCACAAGTAGAGTGATCGAAAGATGAAAAGCACTTTGGAAAGAGAGTTAAACAG  839
                ||||||||||||||||||||||||||||||||||||||||||||||||||||||||||||
Sbjct  2402378  GATAGCGCACAAGTAGAGTGATCGAAAGATGAAAAGCACTTTGGAAAGAGAGTTAAACAG  2402437

Query  840      TACGTGAAATTGTTGAAAGGGAAGCGCTTGCAATCAGACTTGCACTTGGTGTTCATCAGG  899
                ||||||||||||||||||||||||||||||||||||||||||||||||||||||||||||
Sbjct  2402438  TACGTGAAATTGTTGAAAGGGAAGCGCTTGCAATCAGACTTGCACTTGGTGTTCATCAGG  2402497

Query  900      GTTTCGTGCCCTGTGTACTTCATCAAGTTCAGGCCAGCATCAGTTTGAGTGGTTAGATAA  959
                ||||||||||||||||||||||||||||||||||||||||||||||||||||||||||||
Sbjct  2402498  GTTTCGTGCCCTGTGTACTTCATCAAGTTCAGGCCAGCATCAGTTTGAGTGGTTAGATAA  2402557

Query  960      AGGCTTGGAGAATGTGGCCCTCTTCGGGGGGTGTTATAGCTCCAGGTGCAATGTAGCCTA  1019
                ||||||||||||||||||||||||||||||||||||||||||||||||||||||||||||
Sbjct  2402558  AGGCTTGGAGAATGTGGCCCTCTTCGGGGGGTGTTATAGCTCCAGGTGCAATGTAGCCTA  2402617

Query  1020     CTTGGACTGAGGACCGCGCTTCGGCTAGGATGCTGGCGTAATGGTTGTAAGCGAC  1074
                |||||||||||||||||||||||||||||||||||||||||||||||||||||||
Sbjct  2402618  CTTGGACTGAGGACCGCGCTTCGGCTAGGATGCTGGCGTAATGGTTGTAAGCGAC  2402672


 Score = 1978 bits (1071),  Expect = 0.0
 Identities = 1074/1075 (99%), Gaps = 1/1075 (0%)
 Strand=Plus/Plus

Query  1        CCCG-AAGGGTAGACCTCCCACCCTTGTGTATTATTACTTTGTTGCTTTGGCGAGCTGCT  59
                |||| |||||||||||||||||||||||||||||||||||||||||||||||||||||||
Sbjct  2412076  CCCGAAAGGGTAGACCTCCCACCCTTGTGTATTATTACTTTGTTGCTTTGGCGAGCTGCT  2412135

Query  60       CTTCGGGGCCTTGTATGCTCGCCAGAGAATATCAAAACTCTTTTTATTAATGTCGTCTGA  119
                ||||||||||||||||||||||||||||||||||||||||||||||||||||||||||||
Sbjct  2412136  CTTCGGGGCCTTGTATGCTCGCCAGAGAATATCAAAACTCTTTTTATTAATGTCGTCTGA  2412195

Query  120      GTACTATATAATAGTTAAAACTTTCAACAACGGATCTCTTGGTTCTGGCATCGATGAAGA  179
                ||||||||||||||||||||||||||||||||||||||||||||||||||||||||||||
Sbjct  2412196  GTACTATATAATAGTTAAAACTTTCAACAACGGATCTCTTGGTTCTGGCATCGATGAAGA  2412255

Query  180      ACGCAGCGAAATGCGATAAGTAATGTGAATTGCAGAATTCAGTGAATCATCGAATCTTTG  239
                ||||||||||||||||||||||||||||||||||||||||||||||||||||||||||||
Sbjct  2412256  ACGCAGCGAAATGCGATAAGTAATGTGAATTGCAGAATTCAGTGAATCATCGAATCTTTG  2412315

Query  240      AACGCACATTGCGCCCCTTGGTATTCCGGGGGGCATGCCTGTTCGAGCGTCATTTCAACC  299
                ||||||||||||||||||||||||||||||||||||||||||||||||||||||||||||
Sbjct  2412316  AACGCACATTGCGCCCCTTGGTATTCCGGGGGGCATGCCTGTTCGAGCGTCATTTCAACC  2412375
```

```
Query  300      CTCAAGCTCAGCTTGGTATTGAGTCCATGTCAGTAATGGCAGGCTCTAAAATCAGTGGCG  359
                ||||||||||||||||||||||||||||||||||||||||||||||||||||||||||||
Sbjct  2412376  CTCAAGCTCAGCTTGGTATTGAGTCCATGTCAGTAATGGCAGGCTCTAAAATCAGTGGCG  2412435

Query  360      GCGCCGCTGGGTCCTGAACGTAGTAATATCTCTCGTTACAGGTTCTCGGTGTGCTTCTGC  419
                ||||||||||||||||||||||||||||||||||||||||||||||||||||||||||||
Sbjct  2412436  GCGCCGCTGGGTCCTGAACGTAGTAATATCTCTCGTTACAGGTTCTCGGTGTGCTTCTGC  2412495

Query  420      CAAAACCCAAATTTTCTATGGTTGACCTCGGATCAGGTAGGGATACCCGCTGAACTTAAG  479
                ||||||||||||||||||||||||||||||||||||||||||||||||||||||||||||
Sbjct  2412496  CAAAACCCAAATTTTCTATGGTTGACCTCGGATCAGGTAGGGATACCCGCTGAACTTAAG  2412555

Query  480      CATATCAATAAGCGGAGGAAAAGAAACCAACAGGGATTACCTCAGTAACGGCGAGTGAAG  539
                ||||||||||||||||||||||||||||||||||||||||||||||||||||||||||||
Sbjct  2412556  CATATCAATAAGCGGAGGAAAAGAAACCAACAGGGATTACCTCAGTAACGGCGAGTGAAG  2412615

Query  540      CGGTAAAAGCTCAAATTTGAAATCTGGCTCTTTCAGAGTCCGAGTTGTAATTTGTAGAAG  599
                ||||||||||||||||||||||||||||||||||||||||||||||||||||||||||||
Sbjct  2412616  CGGTAAAAGCTCAAATTTGAAATCTGGCTCTTTCAGAGTCCGAGTTGTAATTTGTAGAAG  2412675

Query  600      ATGCTTCGGGTGTGGTTCCGGTCTAAGTTCCTTGGAACAGGACGTCATAGAGGGTGAGAA  659
                ||||||||||||||||||||||||||||||||||||||||||||||||||||||||||||
Sbjct  2412676  ATGCTTCGGGTGTGGTTCCGGTCTAAGTTCCTTGGAACAGGACGTCATAGAGGGTGAGAA  2412735

Query  660      TCCCGTATGTGACTGGATACCTATGCTCATGTGAAGCTCTTTCGACGAGTCGAGTTGTTT  719
                ||||||||||||||||||||||||||||||||||||||||||||||||||||||||||||
Sbjct  2412736  TCCCGTATGTGACTGGATACCTATGCTCATGTGAAGCTCTTTCGACGAGTCGAGTTGTTT  2412795

Query  720      GGGAATGCAGCTCAAAATGGGTGGTATATTTCATCTAAAGCTAAATATTGGCCAGAGACC  779
                ||||||||||||||||||||||||||||||||||||||||||||||||||||||||||||
Sbjct  2412796  GGGAATGCAGCTCAAAATGGGTGGTATATTTCATCTAAAGCTAAATATTGGCCAGAGACC  2412855

Query  780      GATAGCGCACAAGTAGAGTGATCGAAAGATGAAAAGCACTTTGGAAAGAGAGTTAAACAG  839
                ||||||||||||||||||||||||||||||||||||||||||||||||||||||||||||
Sbjct  2412856  GATAGCGCACAAGTAGAGTGATCGAAAGATGAAAAGCACTTTGGAAAGAGAGTTAAACAG  2412915

Query  840      TACGTGAAATTGTTGAAAGGGAAGCGCTTGCAATCAGACTTGCACTTGGTGTTCATCAGG  899
                ||||||||||||||||||||||||||||||||||||||||||||||||||||||||||||
Sbjct  2412916  TACGTGAAATTGTTGAAAGGGAAGCGCTTGCAATCAGACTTGCACTTGGTGTTCATCAGG  2412975

Query  900      GTTTCGTGCCCTGTGTACTTCATCAAGTTCAGGCCAGCATCAGTTTGAGTGGTTAGATAA  959
                ||||||||||||||||||||||||||||||||||||||||||||||||||||||||||||
Sbjct  2412976  GTTTCGTGCCCTGTGTACTTCATCAAGTTCAGGCCAGCATCAGTTTGAGTGGTTAGATAA  2413035

Query  960      AGGCTTGGAGAATGTGGCCCTCTTCGGGGGGTGTTATAGCTCCAGGTGCAATGTAGCCTA  1019
                ||||||||||||||||||||||||||||||||||||||||||||||||||||||||||||
Sbjct  2413036  AGGCTTGGAGAATGTGGCCCTCTTCGGGGGGTGTTATAGCTCCAGGTGCAATGTAGCCTA  2413095

Query  1020     CTTGGACTGAGGACCGCGCTTCGGCTAGGATGCTGGCGTAATGGTTGTAAGCGAC  1074
                |||||||||||||||||||||||||||||||||||||||||||||||||||||||
Sbjct  2413096  CTTGGACTGAGGACCGCGCTTCGGCTAGGATGCTGGCGTAATGGTTGTAAGCGAC  2413150


 Score = 1973 bits (1068),  Expect = 0.0
 Identities = 1073/1075 (99%), Gaps = 2/1075 (0%)
 Strand=Plus/Plus

Query  1        CCCG-AAGGGTAGACCTCCCACCCTTGTGTATTATTACTTTGTTGCTTTGGCGAGCTGCT  59
                |||| |||||||||||||||||||||||||||||||||||||||||||||||||||||||
Sbjct  2391122  CCCGAAAGGGTAGACCTCCCACCCTTGTGTATTATTACTTTGTTGCTTTGGCGAGCTGCT  2391181

Query  60       CTTCGGGGCCTTGTATGCTCGCCAGAGAATATCAAAACTCTTTTTATTAATGTCGTCTGA  119
                ||||||||||||||||||||||||||||||||||||||||||||||||||||||||||||
```

```
Sbjct  2391182  CTTCGGGGCCTTGTATGCTCGCCAGAGAATATCAAAACTCTTTTTATTAATGTCGTCTGA  2391241

Query  120      GTACTATATAATAGTTAAAACTTTCAACAACGGATCTCTTGGTTCTGGCATCGATGAAGA  179
                ||||||||||||||||||||||||||||||||||||||||||||||||||||||||||||
Sbjct  2391242  GTACTATATAATAGTTAAAACTTTCAACAACGGATCTCTTGGTTCTGGCATCGATGAAGA  2391301

Query  180      ACGCAGCGAAATGCGATAAGTAATGTGAATTGCAGAATTCAGTGAATCATCGAATCTTTG  239
                ||||||||||||||||||||||||||||||||||||||||||||||||||||||||||||
Sbjct  2391302  ACGCAGCGAAATGCGATAAGTAATGTGAATTGCAGAATTCAGTGAATCATCGAATCTTTG  2391361

Query  240      AACGCACATTGCGCCCCTTGGTATTCCGGGGGGCATGCCTGTTCGAGCGTCATTTCAACC  299
                ||||||||||||||||||||||||||||||||||||||||||||||||||||||||||||
Sbjct  2391362  AACGCACATTGCGCCCCTTGGTATTCCGGGGGGCATGCCTGTTCGAGCGTCATTTCAACC  2391421

Query  300      CTCAAGCTCAGCTTGGTATTGAGTCCATGTCAGTAATGGCAGGCTCTAAAATCAGTGGCG  359
                ||||||||||||||||||||||||||||||||||||||||||||||||||||||||||||
Sbjct  2391422  CTCAAGCTCAGCTTGGTATTGAGTCCATGTCAGTAATGGCAGGCTCTAAAATCAGTGGCG  2391481

Query  360      GCGCCGCTGGGTCCTGAACGTAGTAATATCTCTCGTTACAGGTTCTCGGTGTGCTTCTGC  419
                ||||||||||||||||||||||||||||||||||||||||||||||||||||||||||||
Sbjct  2391482  GCGCCGCTGGGTCCTGAACGTAGTAATATCTCTCGTTACAGGTTCTCGGTGTGCTTCTGC  2391541

Query  420      CAAAACCCAAATTTTCTATGGTTGACCTCGGATCAGGTAGGGATACCCGCTGAACTTAAG  479
                ||||||||||||||||||||||||||||||||||||||||||||||||||||||||||||
Sbjct  2391542  CAAAACCCAAATTTTCTATGGTTGACCTCGGATCAGGTAGGGATACCCGCTGAACTTAAG  2391601

Query  480      CATATCAATAAGCGGAGGAAAAGAAACCAACAGGGATTACCTCAGTAACGGCGAGTGAAG  539
                ||||||||||||||||||||||||||||||||||||||||||||||||||||||||||||
Sbjct  2391602  CATATCAATAAGCGGAGGAAAAGAAACCAACAGGGATTACCTCAGTAACGGCGAGTGAAG  2391661

Query  540      CGGTAAAAGCTCAAATTTGAAATCTGGCTCTTTCAGAGTCCGAGTTGTAATTTGTAGAAG  599
                ||||||||||||||||||||||||||||||||||||||||||||||||||||||||||||
Sbjct  2391662  CGGTAAAAGCTCAAATTTGAAATCTGGCTCTTTCAGAGTCCGAGTTGTAATTTGTAGAAG  2391721

Query  600      ATGCTTCGGGTGTGGTTCCGGTCTAAGTTCCTTGGAACAGGACGTCATAGAGGGTGAGAA  659
                ||||||||||||||||||||||||||||||||||||||||||||||||||||||||||||
Sbjct  2391722  ATGCTTCGGGTGTGGTTCCGGTCTAAGTTCCTTGGAACAGGACGTCATAGAGGGTGAGAA  2391781

Query  660      TCCCGTATGTGACTGGATACCTATGCTCATGTGAAGCTCTTTCGACGAGTCGAGTTGTTT  719
                ||||||||||||||||||||||||||||||||||||||||||||||||||||||||||||
Sbjct  2391782  TCCCGTATGTGACTGGATACCTATGCTCATGTGAAGCTCTTTCGACGAGTCGAGTTGTTT  2391841

Query  720      GGGAATGCAGCTCAAAATGGGTGGTATATTTCATCTAAAGCTAAATATTGGCCAGAGACC  779
                ||||||||||||||||||||||||||||||||||||||||||||||||||||||||||||
Sbjct  2391842  GGGAATGCAGCTCAAAATGGGTGGTATATTTCATCTAAAGCTAAATATTGGCCAGAGACC  2391901

Query  780      GATAGCGCACAAGTAGAGTGATCGAAAGATGAAAAGCACTTTGGAAAGAGAGTTAAACAG  839
                ||||||||||||||||||||||||||||||||||||||||||||||||||||||||||||
Sbjct  2391902  GATAGCGCACAAGTAGAGTGATCGAAAGATGAAAAGCACTTTGGAAAGAGAGTTAAACAG  2391961

Query  840      TACGTGAAATTGTTGAAAGGGAAGCGCTTGCAATCAGACTTGCACTTGGTGTTCATCAGG  899
                ||||||||||||||||||||||||||||||||||||||||||||||||||||||||||||
Sbjct  2391962  TACGTGAAATTGTTGAAAGGGAAGCGCTTGCAATCAGACTTGCACTTGGTGTTCATCAGG  2392021

Query  900      GTTTCGTGCCCTGTGTACTTCATCAAGTTCAGGCCAGCATCAGTTTGAGTGGTTAGATAA  959
                ||||||||||||||||||||||||||||||||||||||||||||||||||||||||||||
Sbjct  2392022  GTTTCGTGCCCTGTGTACTTCATCAAGTTCAGGCCAGCATCAGTTTGAGTGGTTAGATAA  2392081

Query  960      AGGCTTGGAGAATGTGGCCCTCTTCGGGGGGTGTTATAGCTCCAGGTGCAATGTAGCCTA  1019
                ||||||||||||||||||||||||| ||||||||||||||||||||||||||||||||||
Sbjct  2392082  AGGCTTGGAGAATGTGGCCCTCTTC-GGGGGTGTTATAGCTCCAGGTGCAATGTAGCCTA  2392140

Query  1020     CTTGGACTGAGGACCGCGCTTCGGCTAGGATGCTGGCGTAATGGTTGTAAGCGAC  1074
                |||||||||||||||||||||||||||||||||||||||||||||||||||||||
```

```
Sbjct  2392141  CTTGGACTGAGGACCGCGCTTCGGCTAGGATGCTGGCGTAATGGTTGTAAGCGAC  2392195


 Score = 1938 bits (1049),  Expect = 0.0
 Identities = 1069/1077 (99%), Gaps = 8/1077 (1%)
 Strand=Plus/Plus

Query  1        CCCG-AAGGGTAGACCTCCCACCCTTGTGTATTATTACTTTGTTGCTTTGGCGAGCTGCT  59
                |||| ||||||||||||||||||||||||||||||||| |||||||||||||||||||||
Sbjct  2422534  CCCGAAAGGGTAGACCTCCCACCCTTGTGTATTATTAC-TTGTTGCTTTGGCGAGCTGCT  2422592

Query  60       CTTCGGGGCCTTGTATGCTCGCCAGAGAATATCAAAACTCTTTTTATTAATGTCGTCTGA  119
                |||| |||||||||||||||||||||||||||||||||||||||||||||||||||||||
Sbjct  2422593  CTTC-GGGCCTTGTATGCTCGCCAGAGAATATCAAAACTCTTTTTATTAATGTCGTCTGA  2422651

Query  120      GTACTATATAATAGTTAAAACTTTCAACAACGGATCTCTTGGTTCTGGCATCGATGAAGA  179
                |||||||||||||||| |||||||||||||||||||||||||||||||||||||||||||
Sbjct  2422652  GTACTATATAATAGTT-AAACTTTCAACAACGGATCTCTTGGTTCTGGCATCGATGAAGA  2422710

Query  180      ACGCAGCGAAATGCGATAAGTAATGTGAATTGCAGAATTCAGTGAATCATCGAATCTTTG  239
                ||||||||||||||||||||||||||||||||||||||||||||||||||||||||||||
Sbjct  2422711  ACGCAGCGAAATGCGATAAGTAATGTGAATTGCAGAATTCAGTGAATCATCGAATCTTTG  2422770

Query  240      AACGCACATTGCGCCCCTTGGTATTCCGGGGGGCATGCCTGTTCGAGCGTCATTTCAACC  299
                ||||||||||||||||||||||||||| ||||||||||||||||||||||||||||||||
Sbjct  2422771  AACGCACATTGCGCCCCTTGGTATTCC-GGGGGCATGCCTGTTCGAGCGTCATTTCAACC  2422829

Query  300      CTCAAGCTCAGCTTGGTATTGAGTCCATGTCAGTAATGGCAGGCTCTAAAATCAGTGGCG  359
                ||||||||||||||||||||||||||||||||||||||||||||||||||||||||||||
Sbjct  2422830  CTCAAGCTCAGCTTGGTATTGAGTCCATGTCAGTAATGGCAGGCTCTAAAATCAGTGGCG  2422889

Query  360      GCGCCGCTGGGTCCTGAACGTAGTAATATCTCTCGTTACAGGTTCTCGGTGTGCTTCTGC  419
                ||||||||||||||||||||||||||||||||||||||||||||||||||||||||||||
Sbjct  2422890  GCGCCGCTGGGTCCTGAACGTAGTAATATCTCTCGTTACAGGTTCTCGGTGTGCTTCTGC  2422949

Query  420      CAAAACCCAAATTTTCTATGGTTGACCTCGGATCAGGTAGGGATACCCG-CTGAACTTAA  478
                ||||||||||||||||||||||||||||||||||||||||||||||||| ||||||||||
Sbjct  2422950  CAAAACCCAAATTTTCTATGGTTGACCTCGGATCAGGTAGGGATACCCGCCTGAACTTAA  2423009

Query  479      GCATATCAATAAGCGGAGGAAAAGAAACCAACAGGGATTACCTCAGTAACGGCGAGTGAA  538
                ||||||||||||||||||||||||||||||||||||||||||||||||||||||||||||
Sbjct  2423010  GCATATCAATAAGCGGAGGAAAAGAAACCAACAGGGATTACCTCAGTAACGGCGAGTGAA  2423069

Query  539      GCGGTAAAAGCTCAAATTTGAAATCTGGCTCTTTCAGAGTCCGAGTTGTAATTTGTAGAA  598
                ||||||||||||||||||||||||||||||||||||||||||||||||||||||||||||
Sbjct  2423070  GCGGTAAAAGCTCAAATTTGAAATCTGGCTCTTTCAGAGTCCGAGTTGTAATTTGTAGAA  2423129

Query  599      GATGCTTCGGGTGTGGTTCCGGTCTAAGTTCCTTGGAACAGGACGTCATAGAGGGTGAGA  658
                ||||||||||||||||||||||||||||||||||||||||||||||||||||||||||||
Sbjct  2423130  GATGCTTCGGGTGTGGTTCCGGTCTAAGTTCCTTGGAACAGGACGTCATAGAGGGTGAGA  2423189

Query  659      ATCCCGTATGTGACTGGATACCTATGCTCAT-GTGAAGCTCTTTCGACGAGTCGAGTTGT  717
                || |||||||||||||||||||||||||||| ||||||||||||||||||||||||||||
Sbjct  2423190  AT-CCGTATGTGACTGGATACCTATGCTCATGGTGAAGCTCTTTCGACGAGTCGAGTTGT  2423248

Query  718      TTGGGAATGCAGCTCAAAATGGGTGGTATATTTCATCTAAAGCTAAATATTGGCCAGAGA  777
                ||||||||||||||||||||||||||||||||||||||||||||||||||||||||||||
Sbjct  2423249  TTGGGAATGCAGCTCAAAATGGGTGGTATATTTCATCTAAAGCTAAATATTGGCCAGAGA  2423308

Query  778      CCGATAGCGCACAAGTAGAGTGATCGAAAGATGAAAAGCACTTTGGAAAGAGAGTTAAAC  837
                ||||||||||||||||||||||||||||||||||||||||||||||||||||||||||||
Sbjct  2423309  CCGATAGCGCACAAGTAGAGTGATCGAAAGATGAAAAGCACTTTGGAAAGAGAGTTAAAC  2423368

Query  838      AGTACGTGAAATTGTTGAAAGGGAAGCGCTTGCAATCAGACTTGCACTTGGTGTTCATCA  897
```

```
                 ||||||||||||||||||||||||||||||||||||||||||||||||||||||||||||
Sbjct  2423369   AGTACGTGAAATTGTTGAAAGGGAAGCGCTTGCAATCAGACTTGCACTTGGTGTTCATCA  2423428

Query  898       GGGTTTCGTGCCCTGTGTACTTCATCAAGTTCAGGCCAGCATCAGTTTGAGTGGTTAGAT  957
                 ||||||||||||||||||||||||||||||||||||||||||||||||||||||||||||
Sbjct  2423429   GGGTTTCGTGCCCTGTGTACTTCATCAAGTTCAGGCCAGCATCAGTTTGAGTGGTTAGAT  2423488

Query  958       AAAGGCTTGGAGAATGTGGCCCTCTTCGGGGGGTGTTATAGCTCCAGGTGCAATGTAGCC  1017
                 ||||||||||||||||||||||||||||||||||||||||||||||||||||||||||||
Sbjct  2423489   AAAGGCTTGGAGAATGTGGCCCTCTTCGGGGGGTGTTATAGCTCCAGGTGCAATGTAGCC  2423548

Query  1018      TACTTGGACTGAGGACCGCGCTTCGGCTAGGATGCTGGCGTAATGGTTGTAAGCGAC  1074
                 |||||||||||||||||||||||||||||||||||||||||||||||||||||||||
Sbjct  2423549   TACTTGGACTGAGGACCGCGCTTCGGCTAGGATGCTGGCGTAATGGTTGTAAGCGAC  2423605


 Score = 1929 bits (1044),  Expect = 0.0
 Identities = 1064/1072 (99%), Gaps = 8/1072 (1%)
 Strand=Plus/Plus

Query  5         AAGGGTAGACCTCCCACCCTTGTGTATTATTACTTTGTTGCTTTGGCGAGCTGCTCTTCG  64
                 ||||||||||||||||||||||||||||||||||||||||||||||||||||||||||||
Sbjct  2433019   AAGGGTAGACCTCCCACCCTTGTGTATTATTACTTTGTTGCTTTGGCGAGCTGCTCTTCG  2433078

Query  65        GGGCCTTGTATGCTCGCCAGAGAATATCAAAACTCTTTTTATTAATGTCGTCTGAGTA-C  123
                 |||||||||||||||||||||||||||||||||||||||||||||||||||||||||| |
Sbjct  2433079   GGGCCTTGTATGCTCGCCAGAGAATATCAAAACTCTTTTTATTAATGTCGTCTGAGTATC  2433138

Query  124       TATATAATAGTTAAAACTTTCAACAACGGATCTCTTGGTTCTGGCATCGATGAAGAACGC  183
                 ||||| ||||||||||||||||||||||||||||||||||||||||||||||||||||||
Sbjct  2433139   TATAT-ATAGTTAAAACTTTCAACAACGGATCTCTTGGTTCTGGCATCGATGAAGAACGC  2433197

Query  184       AGCGAAATGCGATAAGTAATGTGAATTGCAGAATT-CAGTGAATCATCGAATCTTTGAAC  242
                 ||||||||||||||||||||||||||||||||||| ||||||||||||||||||||||||
Sbjct  2433198   AGCGAAATGCGATAAGTAATGTGAATTGCAGAATTCCAGTGAATCATCGAATCTTTGAAC  2433257

Query  243       GCACATTGCGCCCCTTGGTATTCCGGGGGGCATGCCTGTTCGAGCGTCATTTCAACCCTC  302
                 |||||||||||||||||||||||||||||||||| |||||||||||||||||||||||||
Sbjct  2433258   GCACATTGCGCCCCTTGGTATTCCGGGGGGCATG-CTGTTCGAGCGTCATTTCAACCCTC  2433316

Query  303       AAGCTCAGCTTGGTATTGAGTCCATGTCAGTAATGGCAGGCTCTAAAATCAGTGGCGGCG  362
                 ||||||||||||||||||||||||||||||||||||||||||||||||||||||||||||
Sbjct  2433317   AAGCTCAGCTTGGTATTGAGTCCATGTCAGTAATGGCAGGCTCTAAAATCAGTGGCGGCG  2433376

Query  363       CCGCTGGGTCCTGAACGTAGTAATATCTCTCGTTACAGGTTCTCGGTGTGCTTCTGCCAA  422
                 ||||||||||||||||||||||||||||||||||||||||||||||||||||||||||||
Sbjct  2433377   CCGCTGGGTCCTGAACGTAGTAATATCTCTCGTTACAGGTTCTCGGTGTGCTTCTGCCAA  2433436

Query  423       AACCCAAATTTTCTATGGTTGACCTCGGATCAGGTAGGGATACCCGCTGAACTTAAGCAT  482
                 ||||||||||||||||||||||||||||||||||||||||||||||||||||||||||||
Sbjct  2433437   AACCCAAATTTTCTATGGTTGACCTCGGATCAGGTAGGGATACCCGCTGAACTTAAGCAT  2433496

Query  483       ATCAATAAGCGGAGGAAAAGAAACCAACAGGGATTACCTCAGTAACGGCGAGTGAAGCGG  542
                 ||||||||||||||||||||||||||||||||||||||||||||||||||||||||||||
Sbjct  2433497   ATCAATAAGCGGAGGAAAAGAAACCAACAGGGATTACCTCAGTAACGGCGAGTGAAGCGG  2433556

Query  543       TAAAAGCTCAAATTTGAAATCTGGCTCTTTCAGAGTCCGAGTTGTAATTTGTAGAAGATG  602
                 ||||||||||||||||||||||||||||||||||||||||||||||||||||||||||||
Sbjct  2433557   TAAAAGCTCAAATTTGAAATCTGGCTCTTTCAGAGTCCGAGTTGTAATTTGTAGAAGATG  2433616

Query  603       CTTCGGGTGTGGTTCCGGTCTAAGTTCCTTGGAACAGGACGTCATAGAGGGTGAGAATCC  662
                 ||||||||||||||||||||||||||||||||||||||||||||||||||||||||||||
Sbjct  2433617   CTTCGGGTGTGGTTCCGGTCTAAGTTCCTTGGAACAGGACGTCATAGAGGGTGAGAATCC  2433676
```

```
Query  663      CGTATGTGACTGGATACCTATGCTCATGTGAAGCTCTTTCGACGAGTCGAGTTGTTTGGG  722
                ||||||||||||||||||||||||||||||||||||||||||||||||||||||||||||
Sbjct  2433677  CGTATGTGACTGGATACCTATGCTCATGTGAAGCTCTTTCGACGAGTCGAGTTGTTTGGG  2433736

Query  723      AATGCAGCTCAAAATGGGTGGTATATTTCATCTAAAGCTAAATATTGGCCAGAGACCGAT  782
                ||||||||||||| |||||||  |||||||||| ||||||||||||||||||||||||||
Sbjct  2433737  AATGCAGCTCAAA-TGGGTGG--TATTTCATCT-AAGCTAAATATTGGCCAGAGACCGAT  2433792

Query  783      AGCGCACAAGTAGAGTGATCGAAAGATGAAAAGCACTTTGGAAAGAGAGTTAAACAGTAC  842
                ||||||||||||||||||||||||||||||||||||||||||||||||||||||||||||
Sbjct  2433793  AGCGCACAAGTAGAGTGATCGAAAGATGAAAAGCACTTTGGAAAGAGAGTTAAACAGTAC  2433852

Query  843      GTGAAATTGTTGAAAGGGAAGCGCTTGCAATCAGACTTGCACTTGGTGTTCATCAGGGTT  902
                ||||||||||||||||||||||||||||||||||||||||||||||||||||||||||||
Sbjct  2433853  GTGAAATTGTTGAAAGGGAAGCGCTTGCAATCAGACTTGCACTTGGTGTTCATCAGGGTT  2433912

Query  903      TCGTGCCCTGTGTACTTCATCAAGTTCAGGCCAGCATCAGTTTGAGTGGTTAGATAAAGG  962
                ||||||||||||||||||||||||||||||||||||||||||||||||||||||||||||
Sbjct  2433913  TCGTGCCCTGTGTACTTCATCAAGTTCAGGCCAGCATCAGTTTGAGTGGTTAGATAAAGG  2433972

Query  963      CTTGGAGAATGTGGCCCTCTTCGGGGGGTGTTATAGCTCCAGGTGCAATGTAGCCTACTT  1022
                ||||||||||||||||||||||||||||||||||||||||||||||||||||||||||||
Sbjct  2433973  CTTGGAGAATGTGGCCCTCTTCGGGGGGTGTTATAGCTCCAGGTGCAATGTAGCCTACTT  2434032

Query  1023     GGACTGAGGACCGCGCTTCGGCTAGGATGCTGGCGTAATGGTTGTAAGCGAC  1074
                ||||||||||||||||||||||||||||||||||||||||||||||||||||
Sbjct  2434033  GGACTGAGGACCGCGCTTCGGCTAGGATGCTGGCGTAATGGTTGTAAGCGAC  2434084


>KC311494.1 Sclerotinia sclerotiorum isolate KR1121_1 18S ribosomal RNA gene,
partial sequence; internal transcribed spacer 1, 5.8S ribosomal
RNA gene, and internal transcribed spacer 2, complete
sequence; and 28S ribosomal RNA gene, partial sequence
Length=1738

 Score = 1978 bits (1071),  Expect = 0.0
 Identities = 1074/1075 (99%), Gaps = 1/1075 (0%)
 Strand=Plus/Plus

Query  1    CCCG-AAGGGTAGACCTCCCACCCTTGTGTATTATTACTTTGTTGCTTTGGCGAGCTGCT  59
            |||| |||||||||||||||||||||||||||||||||||||||||||||||||||||||
Sbjct  53   CCCGAAAGGGTAGACCTCCCACCCTTGTGTATTATTACTTTGTTGCTTTGGCGAGCTGCT  112

Query  60   CTTCGGGGCCTTGTATGCTCGCCAGAGAATATCAAAACTCTTTTTATTAATGTCGTCTGA  119
            ||||||||||||||||||||||||||||||||||||||||||||||||||||||||||||
Sbjct  113  CTTCGGGGCCTTGTATGCTCGCCAGAGAATATCAAAACTCTTTTTATTAATGTCGTCTGA  172

Query  120  GTACTATATAATAGTTAAAACTTTCAACAACGGATCTCTTGGTTCTGGCATCGATGAAGA  179
            ||||||||||||||||||||||||||||||||||||||||||||||||||||||||||||
Sbjct  173  GTACTATATAATAGTTAAAACTTTCAACAACGGATCTCTTGGTTCTGGCATCGATGAAGA  232

Query  180  ACGCAGCGAAATGCGATAAGTAATGTGAATTGCAGAATTCAGTGAATCATCGAATCTTTG  239
            ||||||||||||||||||||||||||||||||||||||||||||||||||||||||||||
Sbjct  233  ACGCAGCGAAATGCGATAAGTAATGTGAATTGCAGAATTCAGTGAATCATCGAATCTTTG  292

Query  240  AACGCACATTGCGCCCCTTGGTATTCCGGGGGGCATGCCTGTTCGAGCGTCATTTCAACC  299
            ||||||||||||||||||||||||||||||||||||||||||||||||||||||||||||
Sbjct  293  AACGCACATTGCGCCCCTTGGTATTCCGGGGGGCATGCCTGTTCGAGCGTCATTTCAACC  352

Query  300  CTCAAGCTCAGCTTGGTATTGAGTCCATGTCAGTAATGGCAGGCTCTAAAATCAGTGGCG  359
            ||||||||||||||||||||||||||||||||||||||||||||||||||||||||||||
Sbjct  353  CTCAAGCTCAGCTTGGTATTGAGTCCATGTCAGTAATGGCAGGCTCTAAAATCAGTGGCG  412

Query  360  GCGCCGCTGGGTCCTGAACGTAGTAATATCTCTCGTTACAGGTTCTCGGTGTGCTTCTGC  419
```

```
                ||||||||||||||||||||||||||||||||||||||||||||||||||||||||||||
Sbjct  413    GCGCCGCTGGGTCCTGAACGTAGTAATATCTCTCGTTACAGGTTCTCGGTGTGCTTCTGC  472

Query  420    CAAAACCCAAATTTTCTATGGTTGACCTCGGATCAGGTAGGGATACCCGCTGAACTTAAG  479
              ||||||||||||||||||||||||||||||||||||||||||||||||||||||||||||
Sbjct  473    CAAAACCCAAATTTTCTATGGTTGACCTCGGATCAGGTAGGGATACCCGCTGAACTTAAG  532

Query  480    CATATCAATAAGCGGAGGAAAAGAAACCAACAGGGATTACCTCAGTAACGGCGAGTGAAG  539
              ||||||||||||||||||||||||||||||||||||||||||||||||||||||||||||
Sbjct  533    CATATCAATAAGCGGAGGAAAAGAAACCAACAGGGATTACCTCAGTAACGGCGAGTGAAG  592

Query  540    CGGTAAAAGCTCAAATTTGAAATCTGGCTCTTTCAGAGTCCGAGTTGTAATTTGTAGAAG  599
              ||||||||||||||||||||||||||||||||||||||||||||||||||||||||||||
Sbjct  593    CGGTAAAAGCTCAAATTTGAAATCTGGCTCTTTCAGAGTCCGAGTTGTAATTTGTAGAAG  652

Query  600    ATGCTTCGGGTGTGGTTCCGGTCTAAGTTCCTTGGAACAGGACGTCATAGAGGGTGAGAA  659
              ||||||||||||||||||||||||||||||||||||||||||||||||||||||||||||
Sbjct  653    ATGCTTCGGGTGTGGTTCCGGTCTAAGTTCCTTGGAACAGGACGTCATAGAGGGTGAGAA  712

Query  660    TCCCGTATGTGACTGGATACCTATGCTCATGTGAAGCTCTTTCGACGAGTCGAGTTGTTT  719
              ||||||||||||||||||||||||||||||||||||||||||||||||||||||||||||
Sbjct  713    TCCCGTATGTGACTGGATACCTATGCTCATGTGAAGCTCTTTCGACGAGTCGAGTTGTTT  772

Query  720    GGGAATGCAGCTCAAAATGGGTGGTATATTTCATCTAAAGCTAAATATTGGCCAGAGACC  779
              ||||||||||||||||||||||||||||||||||||||||||||||||||||||||||||
Sbjct  773    GGGAATGCAGCTCAAAATGGGTGGTATATTTCATCTAAAGCTAAATATTGGCCAGAGACC  832

Query  780    GATAGCGCACAAGTAGAGTGATCGAAAGATGAAAAGCACTTTGGAAAGAGAGTTAAACAG  839
              ||||||||||||||||||||||||||||||||||||||||||||||||||||||||||||
Sbjct  833    GATAGCGCACAAGTAGAGTGATCGAAAGATGAAAAGCACTTTGGAAAGAGAGTTAAACAG  892

Query  840    TACGTGAAATTGTTGAAAGGGAAGCGCTTGCAATCAGACTTGCACTTGGTGTTCATCAGG  899
              ||||||||||||||||||||||||||||||||||||||||||||||||||||||||||||
Sbjct  893    TACGTGAAATTGTTGAAAGGGAAGCGCTTGCAATCAGACTTGCACTTGGTGTTCATCAGG  952

Query  900    GTTTCGTGCCCTGTGTACTTCATCAAGTTCAGGCCAGCATCAGTTTGAGTGGTTAGATAA  959
              ||||||||||||||||||||||||||||||||||||||||||||||||||||||||||||
Sbjct  953    GTTTCGTGCCCTGTGTACTTCATCAAGTTCAGGCCAGCATCAGTTTGAGTGGTTAGATAA  1012

Query  960    AGGCTTGGAGAATGTGGCCCTCTTCGGGGGGTGTTATAGCTCCAGGTGCAATGTAGCCTA  1019
              ||||||||||||||||||||||||||||||||||||||||||||||||||||||||||||
Sbjct  1013   AGGCTTGGAGAATGTGGCCCTCTTCGGGGGGTGTTATAGCTCCAGGTGCAATGTAGCCTA  1072

Query  1020   CTTGGACTGAGGACCGCGCTTCGGCTAGGATGCTGGCGTAATGGTTGTAAGCGAC  1074
              |||||||||||||||||||||||||||||||||||||||||||||||||||||||
Sbjct  1073   CTTGGACTGAGGACCGCGCTTCGGCTAGGATGCTGGCGTAATGGTTGTAAGCGAC  1127


>MZ048353.1 Sclerotinia subarctica voucher WU 43983 small subunit ribosomal
RNA gene, partial sequence; internal transcribed spacer 1,
5.8S ribosomal RNA gene, and internal transcribed spacer 2,
complete sequence; and large subunit ribosomal RNA gene, partial
sequence
Length=1128

 Score = 1925 bits (1042),  Expect = 0.0
 Identities = 1051/1055 (99%), Gaps = 1/1055 (0%)
 Strand=Plus/Plus

Query  1      CCCG-AAGGGTAGACCTCCCACCCTTGTGTATTATTACTTTGTTGCTTTGGCGAGCTGCT  59
              |||| |||||||||||||||||||||||||||||||||||||||||||||||||||||||
Sbjct  74     CCCGAAAGGGTAGACCTCCCACCCTTGTGTATTATTACTTTGTTGCTTTGGCGAGCTGCT  133

Query  60     CTTCGGGGCCTTGTATGCTCGCCAGAGAATATCAAAACTCTTTTTATTAATGTCGTCTGA  119
```

```
             ||||||||||||||||||||||||||||||||||||||||||||||||||||||||||||
Sbjct  134   CTTCGGGGCCTTGTATGCTCGCCAGAGAATATCAAAACTCTTTTTATTAATGTCGTCTGA  193

Query  120   GTACTATATAATAGTTAAAACTTTCAACAACGGATCTCTTGGTTCTGGCATCGATGAAGA  179
             ||||||||||||||||||||||||||||||||||||||||||||||||||||||||||||
Sbjct  194   GTACTATATAATAGTTAAAACTTTCAACAACGGATCTCTTGGTTCTGGCATCGATGAAGA  253

Query  180   ACGCAGCGAAATGCGATAAGTAATGTGAATTGCAGAATTCAGTGAATCATCGAATCTTTG  239
             ||||||||||||||||||||||||||||||||||||||||||||||||||||||||||||
Sbjct  254   ACGCAGCGAAATGCGATAAGTAATGTGAATTGCAGAATTCAGTGAATCATCGAATCTTTG  313

Query  240   AACGCACATTGCGCCCCTTGGTATTCCGGGGGGCATGCCTGTTCGAGCGTCATTTCAACC  299
             ||||||||||||||||||||||||||||||||||||||||||||||||||||||||||||
Sbjct  314   AACGCACATTGCGCCCCTTGGTATTCCGGGGGGCATGCCTGTTCGAGCGTCATTTCAACC  373

Query  300   CTCAAGCTCAGCTTGGTATTGAGTCCATGTCAGTAATGGCAGGCTCTAAAATCAGTGGCG  359
             ||||||||||||||||||||||||| ||||||| ||||||||||||||||||||||||||
Sbjct  374   CTCAAGCTCAGCTTGGTATTGAGTCTATGTCAGCAATGGCAGGCTCTAAAATCAGTGGCG  433

Query  360   GCGCCGCTGGGTCCTGAACGTAGTAATATCTCTCGTTACAGGTTCTCGGTGTGCTTCTGC  419
             ||||||||||||||||||||||||||||||||||||||||||||||||||||||||||||
Sbjct  434   GCGCCGCTGGGTCCTGAACGTAGTAATATCTCTCGTTACAGGTTCTCGGTGTGCTTCTGC  493

Query  420   CAAAACCCAAATTTTCTATGGTTGACCTCGGATCAGGTAGGGATACCCGCTGAACTTAAG  479
             ||||||||||| ||||||||||||||||||||||||||||||||||||||||||||||||
Sbjct  494   CAAAACCCAAACTTTCTATGGTTGACCTCGGATCAGGTAGGGATACCCGCTGAACTTAAG  553

Query  480   CATATCAATAAGCGGAGGAAAAGAAACCAACAGGGATTACCTCAGTAACGGCGAGTGAAG  539
             ||||||||||||||||||||||||||||||||||||||||||||||||||||||||||||
Sbjct  554   CATATCAATAAGCGGAGGAAAAGAAACCAACAGGGATTACCTCAGTAACGGCGAGTGAAG  613

Query  540   CGGTAAAAGCTCAAATTTGAAATCTGGCTCTTTCAGAGTCCGAGTTGTAATTTGTAGAAG  599
             ||||||||||||||||||||||||||||||||||||||||||||||||||||||||||||
Sbjct  614   CGGTAAAAGCTCAAATTTGAAATCTGGCTCTTTCAGAGTCCGAGTTGTAATTTGTAGAAG  673

Query  600   ATGCTTCGGGTGTGGTTCCGGTCTAAGTTCCTTGGAACAGGACGTCATAGAGGGTGAGAA  659
             ||||||||||||||||||||||||||||||||||||||||||||||||||||||||||||
Sbjct  674   ATGCTTCGGGTGTGGTTCCGGTCTAAGTTCCTTGGAACAGGACGTCATAGAGGGTGAGAA  733

Query  660   TCCCGTATGTGACTGGATACCTATGCTCATGTGAAGCTCTTTCGACGAGTCGAGTTGTTT  719
             ||||||||||||||||||||||||||||||||||||||||||||||||||||||||||||
Sbjct  734   TCCCGTATGTGACTGGATACCTATGCTCATGTGAAGCTCTTTCGACGAGTCGAGTTGTTT  793

Query  720   GGGAATGCAGCTCAAAATGGGTGGTATATTTCATCTAAAGCTAAATATTGGCCAGAGACC  779
             ||||||||||||||||||||||||||||||||||||||||||||||||||||||||||||
Sbjct  794   GGGAATGCAGCTCAAAATGGGTGGTATATTTCATCTAAAGCTAAATATTGGCCAGAGACC  853

Query  780   GATAGCGCACAAGTAGAGTGATCGAAAGATGAAAAGCACTTTGGAAAGAGAGTTAAACAG  839
             ||||||||||||||||||||||||||||||||||||||||||||||||||||||||||||
Sbjct  854   GATAGCGCACAAGTAGAGTGATCGAAAGATGAAAAGCACTTTGGAAAGAGAGTTAAACAG  913

Query  840   TACGTGAAATTGTTGAAAGGGAAGCGCTTGCAATCAGACTTGCACTTGGTGTTCATCAGG  899
             ||||||||||||||||||||||||||||||||||||||||||||||||||||||||||||
Sbjct  914   TACGTGAAATTGTTGAAAGGGAAGCGCTTGCAATCAGACTTGCACTTGGTGTTCATCAGG  973

Query  900   GTTTCGTGCCCTGTGTACTTCATCAAGTTCAGGCCAGCATCAGTTTGAGTGGTTAGATAA  959
             ||||||||||||||||||||||||||||||||||||||||||||||||||||||||||||
Sbjct  974   GTTTCGTGCCCTGTGTACTTCATCAAGTTCAGGCCAGCATCAGTTTGAGTGGTTAGATAA  1033

Query  960   AGGCTTGGAGAATGTGGCCCTCTTCGGGGGGTGTTATAGCTCCAGGTGCAATGTAGCCTA  1019
             ||||||||||||||||||||||||||||||||||||||||||||||||||||||||||||
Sbjct  1034  AGGCTTGGAGAATGTGGCCCTCTTCGGGGGGTGTTATAGCTCCAGGTGCAATGTAGCCTA  1093

Query  1020  CTTGGACTGAGGACCGCGCTTCGGCTAGGATGCTG  1054
```

```
             |||||||||||||||||||||||||||||||||||
Sbjct  1094  CTTGGACTGAGGACCGCGCTTCGGCTAGGATGCTG  1128
```

>MZ048352.1 Stromatinia rapulum voucher WU 43986 small subunit ribosomal RNA gene, partial sequence; internal transcribed spacer 1, 5.8S ribosomal RNA gene, and internal transcribed spacer 2, complete sequence; and large subunit ribosomal RNA gene, partial sequence
Length=1126

```
 Score = 1905 bits (1031),  Expect = 0.0
 Identities = 1046/1053 (99%), Gaps = 1/1053 (0%)
 Strand=Plus/Plus

Query  1     CCCG-AAGGGTAGACCTCCCACCCTTGTGTATTATTACTTTGTTGCTTTGGCGAGCTGCT  59
             |||| ||||||||||||||||||||||||||||||||||||||||||||||||||||||
Sbjct  74    CCCGAAAGGGTAGACCTCCCACCCTTGTGTATTATTACTTTGTTGCTTTGGCGAGCTGCC  133

Query  60    CTTCGGGGCCTTGTATGCTCGCCAGAGAATATCAAAACTCTTTTTATTAATGTCGTCTGA  119
             |||||| |||||||||||||||||||||||||||||||||||||||||||||||||||||
Sbjct  134   CTTCGGAGCCTTGTATGCTCGCCAGAGAATATCAAAACTCTTTTTATTAATGTCGTCTGA  193

Query  120   GTACTATATAATAGTTAAAACTTTCAACAACGGATCTCTTGGTTCTGGCATCGATGAAGA  179
             ||||||||||||||||||||||||||||||||||||||||||||||||||||||||||||
Sbjct  194   GTACTATATAATAGTTAAAACTTTCAACAACGGATCTCTTGGTTCTGGCATCGATGAAGA  253

Query  180   ACGCAGCGAAATGCGATAAGTAATGTGAATTGCAGAATTCAGTGAATCATCGAATCTTTG  239
             ||||||||||||||||||||||||||||||||||||||||||||||||||||||||||||
Sbjct  254   ACGCAGCGAAATGCGATAAGTAATGTGAATTGCAGAATTCAGTGAATCATCGAATCTTTG  313

Query  240   AACGCACATTGCGCCCCTTGGTATTCCGGGGGGCATGCCTGTTCGAGCGTCATTTCAACC  299
             ||||||||||||||||||||||||||||||||||||||||||||||||||||||||||||
Sbjct  314   AACGCACATTGCGCCCCTTGGTATTCCGGGGGGCATGCCTGTTCGAGCGTCATTTCAACC  373

Query  300   CTCAAGCTCAGCTTGGTATTGAGTCCATGTCAGTAATGGCAGGCTCTAAAATCAGTGGCG  359
             ||||||||||||||||||||||||||||||||| ||||||||||||||||||||||||||
Sbjct  374   CTCAAGCTCAGCTTGGTATTGAGTCCATGTCAGCAATGGCAGGCTCTAAAATCAGTGGCG  433

Query  360   GCGCCGCTGGGTCCTGAACGTAGTAATATCTCTCGTTACAGGTTCTCGGTGTGCTTCTGC  419
             |||||||||||||||||||||||||||||||||||||||||||||||||||||||||||
Sbjct  434   GCGCCGCTGGGTCCTGAACGTAGTAATATCTCTCGTTACAGGTTCTCGGTGTGCTTCTGT  493

Query  420   CAAAACCCAAATTTTCTATGGTTGACCTCGGATCAGGTAGGGATACCCGCTGAACTTAAG  479
             ||||||| ||||||||||||||||||||||||||||||||||||||||||||||||||||
Sbjct  494   CAAAACCTAAATTTTCTATGGTTGACCTCGGATCAGGTAGGGATACCCGCTGAACTTAAG  553

Query  480   CATATCAATAAGCGGAGGAAAAGAAACCAACAGGGATTACCTCAGTAACGGCGAGTGAAG  539
             ||||||||||||||||||||||||||||||||||||||||||||||||||||||||||||
Sbjct  554   CATATCAATAAGCGGAGGAAAAGAAACCAACAGGGATTACCTCAGTAACGGCGAGTGAAG  613

Query  540   CGGTAAAAGCTCAAATTTGAAATCTGGCTCTTTCAGAGTCCGAGTTGTAATTTGTAGAAG  599
             ||||||||||||||||||||||||||||||||| ||||||||||||||||||||||||||
Sbjct  614   CGGTAAAAGCTCAAATTTGAAATCTGGCTCTTTTAGAGTCCGAGTTGTAATTTGTAGAAG  673

Query  600   ATGCTTCGGGTGTGGTTCCGGTCTAAGTTCCTTGGAACAGGACGTCATAGAGGGTGAGAA  659
             ||||||||||||||||||||||||||||||||||||||||||||||||||||||||||||
Sbjct  674   ATGCTTCGGGTGTGGTTCCGGTCTAAGTTCCTTGGAACAGGACGTCATAGAGGGTGAGAA  733

Query  660   TCCCGTATGTGACTGGATACCTATGCTCATGTGAAGCTCTTTCGACGAGTCGAGTTGTTT  719
             ||||||||||||||||||||||||||||||||||||||||||||||||||||||||||||
Sbjct  734   TCCCGTATGTGACTGGATACCTATGCTCATGTGAAGCTCTTTCGACGAGTCGAGTTGTTT  793

Query  720   GGGAATGCAGCTCAAAATGGGTGGTATATTTCATCTAAAGCTAAATATTGGCCAGAGACC  779
```

```
              ||||||||||||||||||||||||||||||||||||||||||||||||||||||||||||
Sbjct  794    GGGAATGCAGCTCAAAATGGGTGGTATATTTCATCTAAAGCTAAATATTGGCCAGAGACC  853

Query  780    GATAGCGCACAAGTAGAGTGATCGAAAGATGAAAAGCACTTTGGAAAGAGAGTTAAACAG  839
              ||||||||||||||||||||||||||||||||||||||||||||||||||||||||||||
Sbjct  854    GATAGCGCACAAGTAGAGTGATCGAAAGATGAAAAGCACTTTGGAAAGAGAGTTAAACAG  913

Query  840    TACGTGAAATTGTTGAAAGGGAAGCGCTTGCAATCAGACTTGCACTTGGTGTTCATCAGG  899
              ||||||||||||||||||||||||||||||||||||||||||||||||||||||||||||
Sbjct  914    TACGTGAAATTGTTGAAAGGGAAGCGCTTGCAATCAGACTTGCACTTGGTGTTCATCAGG  973

Query  900    GTTTCGTGCCCTGTGTACTTCATCAAGTTCAGGCCAGCATCAGTTTGAGTGGTTAGATAA  959
              ||||||||||||||||||||||||||||||||||||||||||||||||||||||||||||
Sbjct  974    GTTTCGTGCCCTGTGTACTTCATCAAGTTCAGGCCAGCATCAGTTTGAGTGGTTAGATAA  1033

Query  960    AGGCTTGGAGAATGTGGCCCTCTTCGGGGGGTGTTATAGCTCCAGGTGCAATGTAGCCTA  1019
              ||||||||||||||||||||||||||||||||||||||||||||||||||||||||||||
Sbjct  1034   AGGCTTGGAGAATGTGGCCCTCTTCGGGGGGTGTTATAGCTCCAGGTGCAATGTAGCCTA  1093

Query  1020   CTTGGACTGAGGACCGCGCTTCGGCTAGGATGC  1052
              |||||||||||||||||||||||||||||||||
Sbjct  1094   CTTGGACTGAGGACCGCGCTTCGGCTAGGATGC  1126


>KR094468.1 Botrytis cinerea strain G409 18S ribosomal RNA gene, partial
sequence; internal transcribed spacer 1, 5.8S ribosomal RNA
gene, and internal transcribed spacer 2, complete sequence;
and 28S ribosomal RNA gene, partial sequence
Length=1162

 Score = 1901 bits (1029),  Expect = 0.0
 Identities = 1061/1076 (99%), Gaps = 4/1076 (0%)
 Strand=Plus/Plus

Query  1      CCCG-AAGGGTAGACCTCCCACCCTTGTGTATTATTACTTTGTTGCTTTGGCGAGCTGCT  59
              |||| |||||||||||||||||||||||||||||||||||||||||||||||||||||| 
Sbjct  70     CCCGAAAGGGTAGACCTCCCACCCTTGTGTATTATTACTTTGTTGCTTTGGCGAGCTGC-  128

Query  60     CTTCGGGGCCTTGTATGCTCGCCAGAGAATATCAAAACTCTTTTTATTAATGTCGTCTGA  119
              |||| |||||||||||||||||||||||||| ||||||||||||||||||||||||||||
Sbjct  129    CTTC-GGGCCTTGTATGCTCGCCAGAGAATACCAAAACTCTTTTTATTAATGTCGTCTGA  187

Query  120    GTACTATATAATAGTTAAAACTTTCAACAACGGATCTCTTGGTTCTGGCATCGATGAAGA  179
              ||||||||||||||||||||||||||||||||||||||||||||||||||||||||||||
Sbjct  188    GTACTATATAATAGTTAAAACTTTCAACAACGGATCTCTTGGTTCTGGCATCGATGAAGA  247

Query  180    ACGCAGCGAAATGCGATAAGTAATGTGAATTGCAGAATTCAGTGAATCATCGAATCTTTG  239
              ||||||||||||||||||||||||||||||||||||||||||||||||||||||||||||
Sbjct  248    ACGCAGCGAAATGCGATAAGTAATGTGAATTGCAGAATTCAGTGAATCATCGAATCTTTG  307

Query  240    AACGCACATTGCGCCCCTTGGTATTCCGGGGGGCATGCCTGTTCGAGCGTCATTTCAACC  299
              ||||||||||||||||||||||||||||||||||||||||||||||||||||||||||||
Sbjct  308    AACGCACATTGCGCCCCTTGGTATTCCGGGGGGCATGCCTGTTCGAGCGTCATTTCAACC  367

Query  300    CTCAAGCTCAGCTTGGTATTGAGTCCATGTCAGTAATGGCAGGCTCTAAAATCAGTGGCG  359
              |||||||| |||||||||||||||| ||||||||||||||||||||||||||||||||||
Sbjct  368    CTCAAGCTTAGCTTGGTATTGAGTCTATGTCAGTAATGGCAGGCTCTAAAATCAGTGGCG  427

Query  360    GCGCCGCTGGGTCCTGAACGTAGTAATATCTCTCGTTACAGGTTCTCGGTGTGCTTCTGC  419
              ||||||||||||||||||||||||||||||||||||||||||||||||||||||||||||
Sbjct  428    GCGCCGCTGGGTCCTGAACGTAGTAATATCTCTCGTTACAGGTTCTCGGTGTGCTTCTGC  487

Query  420    CAAAACCCAAA-TTTTCTATGGTTGACCTCGGATCAGGTAGGGATACCCGCTGAACTTAA  478
              ||||||||||| ||||||||||||||||||||||||||||||||||||||||||||||||
```

```
Sbjct  488   CAAAACCCAAATTTTTCTATGGTTGACCTCGGATCAGGTAGGGATACCCGCTGAACTTAA  547

Query  479   GCATATCAATAAGCGGAGGAAAAGAAACCAACAGGGATTACCTCAGTAACGGCGAGTGAA  538
             ||||||||||||||||||||||||||||||||||||||||||||||||||||||||||||
Sbjct  548   GCATATCAATAAGCGGAGGAAAAGAAACCAACAGGGATTACCTCAGTAACGGCGAGTGAA  607

Query  539   GCGGTAAAAGCTCAAATTTGAAATCTGGCTCTTTCAGAGTCCGAGTTGTAATTTGTAGAA  598
             |||||||||||||||||||||||||||||||||| ||||||||| |||||||||||||||
Sbjct  608   GCGGTAAAAGCTCAAATTTGAAATCTGGCTCTTTTAGAGTCCGAATTGTAATTTGTAGAA  667

Query  599   GATGCTTCGGGTGTGGTTCCGGTCTAAGTTCCTTGGAACAGGACGTCATAGAGGGTGAGA  658
             ||||||||||||||||||||||||||||||||||||||||||||||||||||||||||||
Sbjct  668   GATGCTTCGGGTGTGGTTCCGGTCTAAGTTCCTTGGAACAGGACGTCATAGAGGGTGAGA  727

Query  659   ATCCCGTATGTGACTGGATACCTATGCTCATGTGAAGCTCTTTCGACGAGTCGAGTTGTT  718
             ||||||||||||||||||||||||||||||||||||||||||||||||||||||||||||
Sbjct  728   ATCCCGTATGTGACTGGATACCTATGCTCATGTGAAGCTCTTTCGACGAGTCGAGTTGTT  787

Query  719   TGGGAATGCAGCTCAAAATGGGTGGTATATTTCATCTAAAGCTAAATATTGGCCAGAGAC  778
             |||||||||||||||||||||| |||||||||| ||||||||||||||||||||||||||
Sbjct  788   TGGGAATGCAGCTCAAAATGGGAGGTATATTTCTTCTAAAGCTAAATATTGGCCAGAGAC  847

Query  779   CGATAGCGCACAAGTAGAGTGATCGAAAGATGAAAAGCACTTTGGAAAGAGAGTTAAACA  838
             ||||||||||||||||||||||||||||||||||||||||||||||||||||||||||||
Sbjct  848   CGATAGCGCACAAGTAGAGTGATCGAAAGATGAAAAGCACTTTGGAAAGAGAGTTAAACA  907

Query  839   GTACGTGAAATTGTTGAAAGGGAAGCGCTTGCAATCAGACTTGCACTTGGTGTTCATCAG  898
             ||||||||||||||||||||||||||||||||||||||||||||||||||||||||||||
Sbjct  908   GTACGTGAAATTGTTGAAAGGGAAGCGCTTGCAATCAGACTTGCACTTGGTGTTCATCAG  967

Query  899   GGTTTCGTGCCCTGTGTACTTCATCAAGTTCAGGCCAGCATCAGTTTGAGTGGTTAGATA  958
             ||| |||| |||||||||||||||||||||||||||||||||||||||||||||||||||
Sbjct  968   GGTCTCGTACCCTGTGTACTTCATCAAGTTCAGGCCAGCATCAGTTTGAGTGGTTAGATA  1027

Query  959   AAGGCTTGGAGAATGTGGCCCTCTTCGGGGGGTGTTATAGCTCCAGGTGCAATGTAGCCT  1018
             ||||||| |||||||||||||||||||||||||||||||||||| |||||||||||||||
Sbjct  1028  AAGGCTTAGAGAATGTGGCCCTCTTCGGGGGGTGTTATAGCTCTAGGTGCAATGTAGCCT  1087

Query  1019  ACTTGGACTGAGGACCGCGCTTCGGCTAGGATGCTGGCGTAATGGTTGTAAGCGAC  1074
             ||||||||||||||||||||||||||||||||||||||||||||||||||||||||
Sbjct  1088  ACTTGGACTGAGGACCGCGCTTCGGCTAGGATGCTGGCGTAATGGTTGTAAGCGAC  1143


>CP009808.1 Botrytis cinerea B05.10 chromosome BCIN04, complete sequence
Length=2468882

 Score = 1901 bits (1029),  Expect = 0.0
 Identities = 1061/1076 (99%), Gaps = 4/1076 (0%)
 Strand=Plus/Minus

Query  1     CCCG-AAGGGTAGACCTCCCACCCTTGTGTATTATTACTTTGTTGCTTTGGCGAGCTGCT  59
             |||| ||||||||||||||||||||||||||||||||||||||||||||||||||||||
Sbjct  4904  CCCGAAAGGGTAGACCTCCCACCCTTGTGTATTATTACTTTGTTGCTTTGGCGAGCTGC-  4846

Query  60    CTTCGGGGCCTTGTATGCTCGCCAGAGAATATCAAAACTCTTTTTATTAATGTCGTCTGA  119
             |||| |||||||||||||||||||||||||| ||||||||||||||||||||||||||||
Sbjct  4845  CTTC-GGGCCTTGTATGCTCGCCAGAGAATACCAAAACTCTTTTTATTAATGTCGTCTGA  4787

Query  120   GTACTATATAATAGTTAAAACTTTCAACAACGGATCTCTTGGTTCTGGCATCGATGAAGA  179
             ||||||||||||||||||||||||||||||||||||||||||||||||||||||||||||
Sbjct  4786  GTACTATATAATAGTTAAAACTTTCAACAACGGATCTCTTGGTTCTGGCATCGATGAAGA  4727

Query  180   ACGCAGCGAAATGCGATAAGTAATGTGAATTGCAGAATTCAGTGAATCATCGAATCTTTG  239
             ||||||||||||||||||||||||||||||||||||||||||||||||||||||||||||
```

```
Sbjct  4726  ACGCAGCGAAATGCGATAAGTAATGTGAATTGCAGAATTCAGTGAATCATCGAATCTTTG  4667

Query  240   AACGCACATTGCGCCCCTTGGTATTCCGGGGGGCATGCCTGTTCGAGCGTCATTTCAACC  299
             ||||||||||||||||||||||||||||||||||||||||||||||||||||||||||||
Sbjct  4666  AACGCACATTGCGCCCCTTGGTATTCCGGGGGGCATGCCTGTTCGAGCGTCATTTCAACC  4607

Query  300   CTCAAGCTCAGCTTGGTATTGAGTCCATGTCAGTAATGGCAGGCTCTAAAATCAGTGGCG  359
             |||||||| |||||||||||||||| ||||||||||||||||||||||||||||||||||
Sbjct  4606  CTCAAGCTTAGCTTGGTATTGAGTCTATGTCAGTAATGGCAGGCTCTAAAATCAGTGGCG  4547

Query  360   GCGCCGCTGGGTCCTGAACGTAGTAATATCTCTCGTTACAGGTTCTCGGTGTGCTTCTGC  419
             ||||||||||||||||||||||||||||||||||||||||||||||||||||||||||||
Sbjct  4546  GCGCCGCTGGGTCCTGAACGTAGTAATATCTCTCGTTACAGGTTCTCGGTGTGCTTCTGC  4487

Query  420   CAAAACCCAAATTTT-CTATGGTTGACCTCGGATCAGGTAGGGATACCCGCTGAACTTAA  478
             ||||||||||||||| ||||||||||||||||||||||||||||||||||||||||||||
Sbjct  4486  CAAAACCCAAATTTTTCTATGGTTGACCTCGGATCAGGTAGGGATACCCGCTGAACTTAA  4427

Query  479   GCATATCAATAAGCGGAGGAAAAGAAACCAACAGGGATTACCTCAGTAACGGCGAGTGAA  538
             ||||||||||||||||||||||||||||||||||||||||||||||||||||||||||||
Sbjct  4426  GCATATCAATAAGCGGAGGAAAAGAAACCAACAGGGATTACCTCAGTAACGGCGAGTGAA  4367

Query  539   GCGGTAAAAGCTCAAATTTGAAATCTGGCTCTTTCAGAGTCCGAGTTGTAATTTGTAGAA  598
             |||||||||||||||||||||||||||||||||| ||||||||| |||||||||||||||
Sbjct  4366  GCGGTAAAAGCTCAAATTTGAAATCTGGCTCTTTTAGAGTCCGAATTGTAATTTGTAGAA  4307

Query  599   GATGCTTCGGGTGTGGTTCCGGTCTAAGTTCCTTGGAACAGGACGTCATAGAGGGTGAGA  658
             ||||||||||||||||||||||||||||||||||||||||||||||||||||||||||||
Sbjct  4306  GATGCTTCGGGTGTGGTTCCGGTCTAAGTTCCTTGGAACAGGACGTCATAGAGGGTGAGA  4247

Query  659   ATCCCGTATGTGACTGGATACCTATGCTCATGTGAAGCTCTTTCGACGAGTCGAGTTGTT  718
             ||||||||||||||||||||||||||||||||||||||||||||||||||||||||||||
Sbjct  4246  ATCCCGTATGTGACTGGATACCTATGCTCATGTGAAGCTCTTTCGACGAGTCGAGTTGTT  4187

Query  719   TGGGAATGCAGCTCAAAATGGGTGGTATATTTCATCTAAAGCTAAATATTGGCCAGAGAC  778
             |||||||||||||||||||||| |||||||||| ||||||||||||||||||||||||||
Sbjct  4186  TGGGAATGCAGCTCAAAATGGGAGGTATATTTCTTCTAAAGCTAAATATTGGCCAGAGAC  4127

Query  779   CGATAGCGCACAAGTAGAGTGATCGAAAGATGAAAAGCACTTTGGAAAGAGAGTTAAACA  838
             ||||||||||||||||||||||||||||||||||||||||||||||||||||||||||||
Sbjct  4126  CGATAGCGCACAAGTAGAGTGATCGAAAGATGAAAAGCACTTTGGAAAGAGAGTTAAACA  4067

Query  839   GTACGTGAAATTGTTGAAAGGGAAGCGCTTGCAATCAGACTTGCACTTGGTGTTCATCAG  898
             ||||||||||||||||||||||||||||||||||||||||||||||||||||||||||||
Sbjct  4066  GTACGTGAAATTGTTGAAAGGGAAGCGCTTGCAATCAGACTTGCACTTGGTGTTCATCAG  4007

Query  899   GGTTTCGTGCCCTGTGTACTTCATCAAGTTCAGGCCAGCATCAGTTTGAGTGGTTAGATA  958
             ||| |||| |||||||||||||||||||||||||||||||||||||||||||||||||||
Sbjct  4006  GGTCTCGTACCCTGTGTACTTCATCAAGTTCAGGCCAGCATCAGTTTGAGTGGTTAGATA  3947

Query  959   AAGGCTTGGAGAATGTGGCCCTCTTCGGGGGGTGTTATAGCTCCAGGTGCAATGTAGCCT  1018
             ||||||| ||||||||||||||||||||||||||||||||||| ||||||||||||||||
Sbjct  3946  AAGGCTTAGAGAATGTGGCCCTCTTCGGGGGGTGTTATAGCTCTAGGTGCAATGTAGCCT  3887

Query  1019  ACTTGGACTGAGGACCGCGCTTCGGCTAGGATGCTGGCGTAATGGTTGTAAGCGAC  1074
             ||||||||||||||||||||||||||||||||||||||||||||||||||||||||
Sbjct  3886  ACTTGGACTGAGGACCGCGCTTCGGCTAGGATGCTGGCGTAATGGTTGTAAGCGAC  3831


>CP080982.1 Botrytis cinerea strain Sl3 chromosome 04
Length=2541613

 Score = 1901 bits (1029),  Expect = 0.0
 Identities = 1061/1076 (99%), Gaps = 4/1076 (0%)
```

```
 Strand=Plus/Minus

Query  1     CCCG-AAGGGTAGACCTCCCACCCTTGTGTATTATTACTTTGTTGCTTTGGCGAGCTGCT  59
             |||| |||||||||||||||||||||||||||||||||||||||||||||||||||||| 
Sbjct  2813  CCCGAAAGGGTAGACCTCCCACCCTTGTGTATTATTACTTTGTTGCTTTGGCGAGCTGC-  2755

Query  60    CTTCGGGGCCTTGTATGCTCGCCAGAGAATATCAAAACTCTTTTTATTAATGTCGTCTGA  119
             |||| |||||||||||||||||||||||||| ||||||||||||||||||||||||||||
Sbjct  2754  CTTC-GGGCCTTGTATGCTCGCCAGAGAATACCAAAACTCTTTTTATTAATGTCGTCTGA  2696

Query  120   GTACTATATAATAGTTAAAACTTTCAACAACGGATCTCTTGGTTCTGGCATCGATGAAGA  179
             ||||||||||||||||||||||||||||||||||||||||||||||||||||||||||||
Sbjct  2695  GTACTATATAATAGTTAAAACTTTCAACAACGGATCTCTTGGTTCTGGCATCGATGAAGA  2636

Query  180   ACGCAGCGAAATGCGATAAGTAATGTGAATTGCAGAATTCAGTGAATCATCGAATCTTTG  239
             ||||||||||||||||||||||||||||||||||||||||||||||||||||||||||||
Sbjct  2635  ACGCAGCGAAATGCGATAAGTAATGTGAATTGCAGAATTCAGTGAATCATCGAATCTTTG  2576

Query  240   AACGCACATTGCGCCCCTTGGTATTCCGGGGGGCATGCCTGTTCGAGCGTCATTTCAACC  299
             ||||||||||||||||||||||||||||||||||||||||||||||||||||||||||||
Sbjct  2575  AACGCACATTGCGCCCCTTGGTATTCCGGGGGGCATGCCTGTTCGAGCGTCATTTCAACC  2516

Query  300   CTCAAGCTCAGCTTGGTATTGAGTCCATGTCAGTAATGGCAGGCTCTAAAATCAGTGGCG  359
             |||||||| |||||||||||||||| ||||||||||||||||||||||||||||||||||
Sbjct  2515  CTCAAGCTTAGCTTGGTATTGAGTCTATGTCAGTAATGGCAGGCTCTAAAATCAGTGGCG  2456

Query  360   GCGCCGCTGGGTCCTGAACGTAGTAATATCTCTCGTTACAGGTTCTCGGTGTGCTTCTGC  419
             ||||||||||||||||||||||||||||||||||||||||||||||||||||||||||||
Sbjct  2455  GCGCCGCTGGGTCCTGAACGTAGTAATATCTCTCGTTACAGGTTCTCGGTGTGCTTCTGC  2396

Query  420   CAAAACCCAAATTTT-CTATGGTTGACCTCGGATCAGGTAGGGATACCCGCTGAACTTAA  478
             ||||||||||||||| ||||||||||||||||||||||||||||||||||||||||||||
Sbjct  2395  CAAAACCCAAATTTTTCTATGGTTGACCTCGGATCAGGTAGGGATACCCGCTGAACTTAA  2336

Query  479   GCATATCAATAAGCGGAGGAAAAGAAACCAACAGGGATTACCTCAGTAACGGCGAGTGAA  538
             ||||||||||||||||||||||||||||||||||||||||||||||||||||||||||||
Sbjct  2335  GCATATCAATAAGCGGAGGAAAAGAAACCAACAGGGATTACCTCAGTAACGGCGAGTGAA  2276

Query  539   GCGGTAAAAGCTCAAATTTGAAATCTGGCTCTTTCAGAGTCCGAGTTGTAATTTGTAGAA  598
             |||||||||||||||||||||||||||||||||| ||||||||| |||||||||||||||
Sbjct  2275  GCGGTAAAAGCTCAAATTTGAAATCTGGCTCTTTTAGAGTCCGAATTGTAATTTGTAGAA  2216

Query  599   GATGCTTCGGGTGTGGTTCCGGTCTAAGTTCCTTGGAACAGGACGTCATAGAGGGTGAGA  658
             ||||||||||||||||||||||||||||||||||||||||||||||||||||||||||||
Sbjct  2215  GATGCTTCGGGTGTGGTTCCGGTCTAAGTTCCTTGGAACAGGACGTCATAGAGGGTGAGA  2156

Query  659   ATCCCGTATGTGACTGGATACCTATGCTCATGTGAAGCTCTTTCGACGAGTCGAGTTGTT  718
             ||||||||||||||||||||||||||||||||||||||||||||||||||||||||||||
Sbjct  2155  ATCCCGTATGTGACTGGATACCTATGCTCATGTGAAGCTCTTTCGACGAGTCGAGTTGTT  2096

Query  719   TGGGAATGCAGCTCAAAATGGGTGGTATATTTCATCTAAAGCTAAATATTGGCCAGAGAC  778
             |||||||||||||||||||||| |||||||||| ||||||||||||||||||||||||||
Sbjct  2095  TGGGAATGCAGCTCAAAATGGGAGGTATATTTCTTCTAAAGCTAAATATTGGCCAGAGAC  2036

Query  779   CGATAGCGCACAAGTAGAGTGATCGAAAGATGAAAAGCACTTTGGAAAGAGAGTTAAACA  838
             ||||||||||||||||||||||||||||||||||||||||||||||||||||||||||||
Sbjct  2035  CGATAGCGCACAAGTAGAGTGATCGAAAGATGAAAAGCACTTTGGAAAGAGAGTTAAACA  1976

Query  839   GTACGTGAAATTGTTGAAAGGGAAGCGCTTGCAATCAGACTTGCACTTGGTGTTCATCAG  898
             ||||||||||||||||||||||||||||||||||||||||||||||||||||||||||||
Sbjct  1975  GTACGTGAAATTGTTGAAAGGGAAGCGCTTGCAATCAGACTTGCACTTGGTGTTCATCAG  1916

Query  899   GGTTTCGTGCCCTGTGTACTTCATCAAGTTCAGGCCAGCATCAGTTTGAGTGGTTAGATA  958
             ||| |||| |||||||||||||||||||||||||||||||||||||||||||||||||||
```

```
Sbjct  1915   GGTCTCGTACCCTGTGTACTTCATCAAGTTCAGGCCAGCATCAGTTTGAGTGGTTAGATA  1856

Query  959    AAGGCTTGGAGAATGTGGCCCTCTTCGGGGGGTGTTATAGCTCCAGGTGCAATGTAGCCT  1018
              ||||||| ||||||||||||||||||||||||||||||||||| ||||||||||||||||
Sbjct  1855   AAGGCTTAGAGAATGTGGCCCTCTTCGGGGGGTGTTATAGCTCTAGGTGCAATGTAGCCT  1796

Query  1019   ACTTGGACTGAGGACCGCGCTTCGGCTAGGATGCTGGCGTAATGGTTGTAAGCGAC  1074
              ||||||||||||||||||||||||||||||||||||||||||||||||||||||||
Sbjct  1795   ACTTGGACTGAGGACCGCGCTTCGGCTAGGATGCTGGCGTAATGGTTGTAAGCGAC  1740


 Score = 1901 bits (1029),  Expect = 0.0
 Identities = 1061/1076 (99%), Gaps = 4/1076 (0%)
 Strand=Plus/Minus

Query  1      CCCG-AAGGGTAGACCTCCCACCCTTGTGTATTATTACTTTGTTGCTTTGGCGAGCTGCT  59
              |||| |||||||||||||||||||||||||||||||||||||||||||||||||||||| 
Sbjct  11792  CCCGAAAGGGTAGACCTCCCACCCTTGTGTATTATTACTTTGTTGCTTTGGCGAGCTGC-  11734

Query  60     CTTCGGGGCCTTGTATGCTCGCCAGAGAATATCAAAACTCTTTTTATTAATGTCGTCTGA  119
              |||| |||||||||||||||||||||||||| ||||||||||||||||||||||||||||
Sbjct  11733  CTTC-GGGCCTTGTATGCTCGCCAGAGAATACCAAAACTCTTTTTATTAATGTCGTCTGA  11675

Query  120    GTACTATATAATAGTTAAAACTTTCAACAACGGATCTCTTGGTTCTGGCATCGATGAAGA  179
              ||||||||||||||||||||||||||||||||||||||||||||||||||||||||||||
Sbjct  11674  GTACTATATAATAGTTAAAACTTTCAACAACGGATCTCTTGGTTCTGGCATCGATGAAGA  11615

Query  180    ACGCAGCGAAATGCGATAAGTAATGTGAATTGCAGAATTCAGTGAATCATCGAATCTTTG  239
              ||||||||||||||||||||||||||||||||||||||||||||||||||||||||||||
Sbjct  11614  ACGCAGCGAAATGCGATAAGTAATGTGAATTGCAGAATTCAGTGAATCATCGAATCTTTG  11555

Query  240    AACGCACATTGCGCCCCTTGGTATTCCGGGGGGCATGCCTGTTCGAGCGTCATTTCAACC  299
              ||||||||||||||||||||||||||||||||||||||||||||||||||||||||||||
Sbjct  11554  AACGCACATTGCGCCCCTTGGTATTCCGGGGGGCATGCCTGTTCGAGCGTCATTTCAACC  11495

Query  300    CTCAAGCTCAGCTTGGTATTGAGTCCATGTCAGTAATGGCAGGCTCTAAAATCAGTGGCG  359
              |||||||| |||||||||||||||| ||||||||||||||||||||||||||||||||||
Sbjct  11494  CTCAAGCTTAGCTTGGTATTGAGTCTATGTCAGTAATGGCAGGCTCTAAAATCAGTGGCG  11435

Query  360    GCGCCGCTGGGTCCTGAACGTAGTAATATCTCTCGTTACAGGTTCTCGGTGTGCTTCTGC  419
              ||||||||||||||||||||||||||||||||||||||||||||||||||||||||||||
Sbjct  11434  GCGCCGCTGGGTCCTGAACGTAGTAATATCTCTCGTTACAGGTTCTCGGTGTGCTTCTGC  11375

Query  420    CAAAACCCAAATTTT-CTATGGTTGACCTCGGATCAGGTAGGGATACCCGCTGAACTTAA  478
              ||||||||||||||| ||||||||||||||||||||||||||||||||||||||||||||
Sbjct  11374  CAAAACCCAAATTTTTCTATGGTTGACCTCGGATCAGGTAGGGATACCCGCTGAACTTAA  11315

Query  479    GCATATCAATAAGCGGAGGAAAAGAAACCAACAGGGATTACCTCAGTAACGGCGAGTGAA  538
              ||||||||||||||||||||||||||||||||||||||||||||||||||||||||||||
Sbjct  11314  GCATATCAATAAGCGGAGGAAAAGAAACCAACAGGGATTACCTCAGTAACGGCGAGTGAA  11255

Query  539    GCGGTAAAAGCTCAAATTTGAAATCTGGCTCTTTCAGAGTCCGAGTTGTAATTTGTAGAA  598
              |||||||||||||||||||||||||||||||||| ||||||||| |||||||||||||||
Sbjct  11254  GCGGTAAAAGCTCAAATTTGAAATCTGGCTCTTTTAGAGTCCGAATTGTAATTTGTAGAA  11195

Query  599    GATGCTTCGGGTGTGGTTCCGGTCTAAGTTCCTTGGAACAGGACGTCATAGAGGGTGAGA  658
              ||||||||||||||||||||||||||||||||||||||||||||||||||||||||||||
Sbjct  11194  GATGCTTCGGGTGTGGTTCCGGTCTAAGTTCCTTGGAACAGGACGTCATAGAGGGTGAGA  11135

Query  659    ATCCCGTATGTGACTGGATACCTATGCTCATGTGAAGCTCTTTCGACGAGTCGAGTTGTT  718
              ||||||||||||||||||||||||||||||||||||||||||||||||||||||||||||
Sbjct  11134  ATCCCGTATGTGACTGGATACCTATGCTCATGTGAAGCTCTTTCGACGAGTCGAGTTGTT  11075

Query  719    TGGGAATGCAGCTCAAAATGGGTGGTATATTTCATCTAAAGCTAAATATTGGCCAGAGAC  778
```

```
                ||||||||||||||||||||||| |||||||||| ||||||||||||||||||||||||||
Sbjct  11074  TGGGAATGCAGCTCAAAATGGGAGGTATATTTCTTCTAAAGCTAAATATTGGCCAGAGAC  11015

Query  779    CGATAGCGCACAAGTAGAGTGATCGAAAGATGAAAAGCACTTTGGAAAGAGAGTTAAACA  838
              ||||||||||||||||||||||||||||||||||||||||||||||||||||||||||||
Sbjct  11014  CGATAGCGCACAAGTAGAGTGATCGAAAGATGAAAAGCACTTTGGAAAGAGAGTTAAACA  10955

Query  839    GTACGTGAAATTGTTGAAAGGGAAGCGCTTGCAATCAGACTTGCACTTGGTGTTCATCAG  898
              ||||||||||||||||||||||||||||||||||||||||||||||||||||||||||||
Sbjct  10954  GTACGTGAAATTGTTGAAAGGGAAGCGCTTGCAATCAGACTTGCACTTGGTGTTCATCAG  10895

Query  899    GGTTTCGTGCCCTGTGTACTTCATCAAGTTCAGGCCAGCATCAGTTTGAGTGGTTAGATA  958
              ||| |||| |||||||||||||||||||||||||||||||||||||||||||||||||||
Sbjct  10894  GGTCTCGTACCCTGTGTACTTCATCAAGTTCAGGCCAGCATCAGTTTGAGTGGTTAGATA  10835

Query  959    AAGGCTTGGAGAATGTGGCCCTCTTCGGGGGGTGTTATAGCTCCAGGTGCAATGTAGCCT  1018
              ||||||| ||||||||||||||||||||||||||||||||||| ||||||||||||||||
Sbjct  10834  AAGGCTTAGAGAATGTGGCCCTCTTCGGGGGGTGTTATAGCTCTAGGTGCAATGTAGCCT  10775

Query  1019   ACTTGGACTGAGGACCGCGCTTCGGCTAGGATGCTGGCGTAATGGTTGTAAGCGAC  1074
              ||||||||||||||||||||||||||||||||||||||||||||||||||||||||
Sbjct  10774  ACTTGGACTGAGGACCGCGCTTCGGCTAGGATGCTGGCGTAATGGTTGTAAGCGAC  10719


>CP080964.1 Botrytis cinerea strain Vv3 chromosome 04
Length=2580065

 Score = 1901 bits (1029),  Expect = 0.0
 Identities = 1061/1076 (99%), Gaps = 4/1076 (0%)
 Strand=Plus/Minus

Query  1     CCCG-AAGGGTAGACCTCCCACCCTTGTGTATTATTACTTTGTTGCTTTGGCGAGCTGCT  59
             |||| ||||||||||||||||||||||||||||||||||||||||||||||||||||||
Sbjct  8516  CCCGAAAGGGTAGACCTCCCACCCTTGTGTATTATTACTTTGTTGCTTTGGCGAGCTGC-  8458

Query  60    CTTCGGGGCCTTGTATGCTCGCCAGAGAATATCAAAACTCTTTTTATTAATGTCGTCTGA  119
             |||| |||||||||||||||||||||||||| ||||||||||||||||||||||||||||
Sbjct  8457  CTTC-GGGCCTTGTATGCTCGCCAGAGAATACCAAAACTCTTTTTATTAATGTCGTCTGA  8399

Query  120   GTACTATATAATAGTTAAAACTTTCAACAACGGATCTCTTGGTTCTGGCATCGATGAAGA  179
             ||||||||||||||||||||||||||||||||||||||||||||||||||||||||||||
Sbjct  8398  GTACTATATAATAGTTAAAACTTTCAACAACGGATCTCTTGGTTCTGGCATCGATGAAGA  8339

Query  180   ACGCAGCGAAATGCGATAAGTAATGTGAATTGCAGAATTCAGTGAATCATCGAATCTTTG  239
             ||||||||||||||||||||||||||||||||||||||||||||||||||||||||||||
Sbjct  8338  ACGCAGCGAAATGCGATAAGTAATGTGAATTGCAGAATTCAGTGAATCATCGAATCTTTG  8279

Query  240   AACGCACATTGCGCCCCTTGGTATTCCGGGGGGCATGCCTGTTCGAGCGTCATTTCAACC  299
             ||||||||||||||||||||||||||||||||||||||||||||||||||||||||||||
Sbjct  8278  AACGCACATTGCGCCCCTTGGTATTCCGGGGGGCATGCCTGTTCGAGCGTCATTTCAACC  8219

Query  300   CTCAAGCTCAGCTTGGTATTGAGTCCATGTCAGTAATGGCAGGCTCTAAAATCAGTGGCG  359
             |||||||| |||||||||||||||| ||||||||||||||||||||||||||||||||||
Sbjct  8218  CTCAAGCTTAGCTTGGTATTGAGTCTATGTCAGTAATGGCAGGCTCTAAAATCAGTGGCG  8159

Query  360   GCGCCGCTGGGTCCTGAACGTAGTAATATCTCTCGTTACAGGTTCTCGGTGTGCTTCTGC  419
             ||||||||||||||||||||||||||||||||||||||||||||||||||||||||||||
Sbjct  8158  GCGCCGCTGGGTCCTGAACGTAGTAATATCTCTCGTTACAGGTTCTCGGTGTGCTTCTGC  8099

Query  420   CAAAACCCAAATTTT-CTATGGTTGACCTCGGATCAGGTAGGGATACCCGCTGAACTTAA  478
             ||||||||||||||| ||||||||||||||||||||||||||||||||||||||||||||
Sbjct  8098  CAAAACCCAAATTTTTCTATGGTTGACCTCGGATCAGGTAGGGATACCCGCTGAACTTAA  8039

Query  479   GCATATCAATAAGCGGAGGAAAAGAAACCAACAGGGATTACCTCAGTAACGGCGAGTGAA  538
```

```
                ||||||||||||||||||||||||||||||||||||||||||||||||||||||||||||
Sbjct  8038   GCATATCAATAAGCGGAGGAAAAGAAACCAACAGGGATTACCTCAGTAACGGCGAGTGAA  7979

Query  539    GCGGTAAAAGCTCAAATTTGAAATCTGGCTCTTTCAGAGTCCGAGTTGTAATTTGTAGAA  598
              |||||||||||||||||||||||||||||||||| ||||||||| |||||||||||||||
Sbjct  7978   GCGGTAAAAGCTCAAATTTGAAATCTGGCTCTTTTAGAGTCCGAATTGTAATTTGTAGAA  7919

Query  599    GATGCTTCGGGTGTGGTTCCGGTCTAAGTTCCTTGGAACAGGACGTCATAGAGGGTGAGA  658
              ||||||||||||||||||||||||||||||||||||||||||||||||||||||||||||
Sbjct  7918   GATGCTTCGGGTGTGGTTCCGGTCTAAGTTCCTTGGAACAGGACGTCATAGAGGGTGAGA  7859

Query  659    ATCCCGTATGTGACTGGATACCTATGCTCATGTGAAGCTCTTTCGACGAGTCGAGTTGTT  718
              ||||||||||||||||||||||||||||||||||||||||||||||||||||||||||||
Sbjct  7858   ATCCCGTATGTGACTGGATACCTATGCTCATGTGAAGCTCTTTCGACGAGTCGAGTTGTT  7799

Query  719    TGGGAATGCAGCTCAAAATGGGTGGTATATTTCATCTAAAGCTAAATATTGGCCAGAGAC  778
              |||||||||||||||||||||| |||||||||| ||||||||||||||||||||||||||
Sbjct  7798   TGGGAATGCAGCTCAAAATGGGAGGTATATTTCTTCTAAAGCTAAATATTGGCCAGAGAC  7739

Query  779    CGATAGCGCACAAGTAGAGTGATCGAAAGATGAAAAGCACTTTGGAAAGAGAGTTAAACA  838
              ||||||||||||||||||||||||||||||||||||||||||||||||||||||||||||
Sbjct  7738   CGATAGCGCACAAGTAGAGTGATCGAAAGATGAAAAGCACTTTGGAAAGAGAGTTAAACA  7679

Query  839    GTACGTGAAATTGTTGAAAGGGAAGCGCTTGCAATCAGACTTGCACTTGGTGTTCATCAG  898
              ||||||||||||||||||||||||||||||||||||||||||||||||||||||||||||
Sbjct  7678   GTACGTGAAATTGTTGAAAGGGAAGCGCTTGCAATCAGACTTGCACTTGGTGTTCATCAG  7619

Query  899    GGTTTCGTGCCCTGTGTACTTCATCAAGTTCAGGCCAGCATCAGTTTGAGTGGTTAGATA  958
              ||| |||| |||||||||||||||||||||||||||||||||||||||||||||||||||
Sbjct  7618   GGTCTCGTACCCTGTGTACTTCATCAAGTTCAGGCCAGCATCAGTTTGAGTGGTTAGATA  7559

Query  959    AAGGCTTGGAGAATGTGGCCCTCTTCGGGGGGTGTTATAGCTCCAGGTGCAATGTAGCCT  1018
              ||||||| ||||||||||||||||||||||||||||||||||| ||||||||||||||||
Sbjct  7558   AAGGCTTAGAGAATGTGGCCCTCTTCGGGGGGTGTTATAGCTCTAGGTGCAATGTAGCCT  7499

Query  1019   ACTTGGACTGAGGACCGCGCTTCGGCTAGGATGCTGGCGTAATGGTTGTAAGCGAC  1074
              ||||||||||||||||||||||||||||||||||||||||||||||||||||||||
Sbjct  7498   ACTTGGACTGAGGACCGCGCTTCGGCTAGGATGCTGGCGTAATGGTTGTAAGCGAC  7443


>MW791983.1 Botrytis cinerea voucher culture Y224P internal transcribed spacer
1, partial sequence; 5.8S ribosomal RNA gene and internal
transcribed spacer 2, complete sequence; and large subunit
ribosomal RNA gene, partial sequence
Length=1364

 Score = 1901 bits (1029),  Expect = 0.0
 Identities = 1061/1076 (99%), Gaps = 4/1076 (0%)
 Strand=Plus/Plus

Query  1      CCCG-AAGGGTAGACCTCCCACCCTTGTGTATTATTACTTTGTTGCTTTGGCGAGCTGCT  59
              |||| |||||||||||||||||||||||||||||||||||||||||||||||||||||| 
Sbjct  20     CCCGAAAGGGTAGACCTCCCACCCTTGTGTATTATTACTTTGTTGCTTTGGCGAGCTGC-  78

Query  60     CTTCGGGGCCTTGTATGCTCGCCAGAGAATATCAAAACTCTTTTTATTAATGTCGTCTGA  119
              |||| |||||||||||||||||||||||||| ||||||||||||||||||||||||||||
Sbjct  79     CTTC-GGGCCTTGTATGCTCGCCAGAGAATACCAAAACTCTTTTTATTAATGTCGTCTGA  137

Query  120    GTACTATATAATAGTTAAAACTTTCAACAACGGATCTCTTGGTTCTGGCATCGATGAAGA  179
              ||||||||||||||||||||||||||||||||||||||||||||||||||||||||||||
Sbjct  138    GTACTATATAATAGTTAAAACTTTCAACAACGGATCTCTTGGTTCTGGCATCGATGAAGA  197

Query  180    ACGCAGCGAAATGCGATAAGTAATGTGAATTGCAGAATTCAGTGAATCATCGAATCTTTG  239
              ||||||||||||||||||||||||||||||||||||||||||||||||||||||||||||
```

```
Sbjct  198   ACGCAGCGAAATGCGATAAGTAATGTGAATTGCAGAATTCAGTGAATCATCGAATCTTTG  257

Query  240   AACGCACATTGCGCCCCTTGGTATTCCGGGGGGCATGCCTGTTCGAGCGTCATTTCAACC  299
             ||||||||||||||||||||||||||||||||||||||||||||||||||||||||||||
Sbjct  258   AACGCACATTGCGCCCCTTGGTATTCCGGGGGGCATGCCTGTTCGAGCGTCATTTCAACC  317

Query  300   CTCAAGCTCAGCTTGGTATTGAGTCCATGTCAGTAATGGCAGGCTCTAAAATCAGTGGCG  359
             |||||||| |||||||||||||||| ||||||||||||||||||||||||||||||||||
Sbjct  318   CTCAAGCTTAGCTTGGTATTGAGTCTATGTCAGTAATGGCAGGCTCTAAAATCAGTGGCG  377

Query  360   GCGCCGCTGGGTCCTGAACGTAGTAATATCTCTCGTTACAGGTTCTCGGTGTGCTTCTGC  419
             ||||||||||||||||||||||||||||||||||||||||||||||||||||||||||||
Sbjct  378   GCGCCGCTGGGTCCTGAACGTAGTAATATCTCTCGTTACAGGTTCTCGGTGTGCTTCTGC  437

Query  420   CAAAACCCAAA-TTTTCTATGGTTGACCTCGGATCAGGTAGGGATACCCGCTGAACTTAA  478
             ||||||||||| ||||||||||||||||||||||||||||||||||||||||||||||||
Sbjct  438   CAAAACCCAAATTTTTCTATGGTTGACCTCGGATCAGGTAGGGATACCCGCTGAACTTAA  497

Query  479   GCATATCAATAAGCGGAGGAAAAGAAACCAACAGGGATTACCTCAGTAACGGCGAGTGAA  538
             ||||||||||||||||||||||||||||||||||||||||||||||||||||||||||||
Sbjct  498   GCATATCAATAAGCGGAGGAAAAGAAACCAACAGGGATTACCTCAGTAACGGCGAGTGAA  557

Query  539   GCGGTAAAAGCTCAAATTTGAAATCTGGCTCTTTCAGAGTCCGAGTTGTAATTTGTAGAA  598
             |||||||||||||||||||||||||||||||||| ||||||||| |||||||||||||||
Sbjct  558   GCGGTAAAAGCTCAAATTTGAAATCTGGCTCTTTTAGAGTCCGAATTGTAATTTGTAGAA  617

Query  599   GATGCTTCGGGTGTGGTTCCGGTCTAAGTTCCTTGGAACAGGACGTCATAGAGGGTGAGA  658
             ||||||||||||||||||||||||||||||||||||||||||||||||||||||||||||
Sbjct  618   GATGCTTCGGGTGTGGTTCCGGTCTAAGTTCCTTGGAACAGGACGTCATAGAGGGTGAGA  677

Query  659   ATCCCGTATGTGACTGGATACCTATGCTCATGTGAAGCTCTTTCGACGAGTCGAGTTGTT  718
             ||||||||||||||||||||||||||||||||||||||||||||||||||||||||||||
Sbjct  678   ATCCCGTATGTGACTGGATACCTATGCTCATGTGAAGCTCTTTCGACGAGTCGAGTTGTT  737

Query  719   TGGGAATGCAGCTCAAAATGGGTGGTATATTTCATCTAAAGCTAAATATTGGCCAGAGAC  778
             |||||||||||||||||||||| |||||||||| ||||||||||||||||||||||||||
Sbjct  738   TGGGAATGCAGCTCAAAATGGGAGGTATATTTCTTCTAAAGCTAAATATTGGCCAGAGAC  797

Query  779   CGATAGCGCACAAGTAGAGTGATCGAAAGATGAAAAGCACTTTGGAAAGAGAGTTAAACA  838
             ||||||||||||||||||||||||||||||||||||||||||||||||||||||||||||
Sbjct  798   CGATAGCGCACAAGTAGAGTGATCGAAAGATGAAAAGCACTTTGGAAAGAGAGTTAAACA  857

Query  839   GTACGTGAAATTGTTGAAAGGGAAGCGCTTGCAATCAGACTTGCACTTGGTGTTCATCAG  898
             ||||||||||||||||||||||||||||||||||||||||||||||||||||||||||||
Sbjct  858   GTACGTGAAATTGTTGAAAGGGAAGCGCTTGCAATCAGACTTGCACTTGGTGTTCATCAG  917

Query  899   GGTTTCGTGCCCTGTGTACTTCATCAAGTTCAGGCCAGCATCAGTTTGAGTGGTTAGATA  958
             ||| |||| |||||||||||||||||||||||||||||||||||||||||||||||||||
Sbjct  918   GGTCTCGTACCCTGTGTACTTCATCAAGTTCAGGCCAGCATCAGTTTGAGTGGTTAGATA  977

Query  959   AAGGCTTGGAGAATGTGGCCCTCTTCGGGGGGTGTTATAGCTCCAGGTGCAATGTAGCCT  1018
             ||||||| ||||||||||||||||||||||||||||||||||| ||||||||||||||||
Sbjct  978   AAGGCTTAGAGAATGTGGCCCTCTTCGGGGGGTGTTATAGCTCTAGGTGCAATGTAGCCT  1037

Query  1019  ACTTGGACTGAGGACCGCGCTTCGGCTAGGATGCTGGCGTAATGGTTGTAAGCGAC  1074
             ||||||||||||||||||||||||||||||||||||||||||||||||||||||||
Sbjct  1038  ACTTGGACTGAGGACCGCGCTTCGGCTAGGATGCTGGCGTAATGGTTGTAAGCGAC  1093
```

```
>MW791982.1 Botrytis cinerea voucher culture Y2240 internal transcribed spacer
1, partial sequence; 5.8S ribosomal RNA gene and internal
transcribed spacer 2, complete sequence; and large subunit
ribosomal RNA gene, partial sequence
Length=1360
```

```
 Score = 1901 bits (1029),  Expect = 0.0
 Identities = 1061/1076 (99%), Gaps = 4/1076 (0%)
 Strand=Plus/Plus

Query  1     CCCG-AAGGGTAGACCTCCCACCCTTGTGTATTATTACTTTGTTGCTTTGGCGAGCTGCT  59
             |||| ||||||||||||||||||||||||||||||||||||||||||||||||||||||
Sbjct  16    CCCGAAAGGGTAGACCTCCCACCCTTGTGTATTATTACTTTGTTGCTTTGGCGAGCTGC-  74

Query  60    CTTCGGGGCCTTGTATGCTCGCCAGAGAATATCAAAACTCTTTTTATTAATGTCGTCTGA  119
             |||| |||||||||||||||||||||||||| ||||||||||||||||||||||||||||
Sbjct  75    CTTC-GGGCCTTGTATGCTCGCCAGAGAATACCAAAACTCTTTTTATTAATGTCGTCTGA  133

Query  120   GTACTATATAATAGTTAAAACTTTCAACAACGGATCTCTTGGTTCTGGCATCGATGAAGA  179
             ||||||||||||||||||||||||||||||||||||||||||||||||||||||||||||
Sbjct  134   GTACTATATAATAGTTAAAACTTTCAACAACGGATCTCTTGGTTCTGGCATCGATGAAGA  193

Query  180   ACGCAGCGAAATGCGATAAGTAATGTGAATTGCAGAATTCAGTGAATCATCGAATCTTTG  239
             ||||||||||||||||||||||||||||||||||||||||||||||||||||||||||||
Sbjct  194   ACGCAGCGAAATGCGATAAGTAATGTGAATTGCAGAATTCAGTGAATCATCGAATCTTTG  253

Query  240   AACGCACATTGCGCCCCTTGGTATTCCGGGGGGCATGCCTGTTCGAGCGTCATTTCAACC  299
             ||||||||||||||||||||||||||||||||||||||||||||||||||||||||||||
Sbjct  254   AACGCACATTGCGCCCCTTGGTATTCCGGGGGGCATGCCTGTTCGAGCGTCATTTCAACC  313

Query  300   CTCAAGCTCAGCTTGGTATTGAGTCCATGTCAGTAATGGCAGGCTCTAAAATCAGTGGCG  359
             |||||||| |||||||||||||||| ||||||||||||||||||||||||||||||||||
Sbjct  314   CTCAAGCTTAGCTTGGTATTGAGTCTATGTCAGTAATGGCAGGCTCTAAAATCAGTGGCG  373

Query  360   GCGCCGCTGGGTCCTGAACGTAGTAATATCTCTCGTTACAGGTTCTCGGTGTGCTTCTGC  419
             ||||||||||||||||||||||||||||||||||||||||||||||||||||||||||||
Sbjct  374   GCGCCGCTGGGTCCTGAACGTAGTAATATCTCTCGTTACAGGTTCTCGGTGTGCTTCTGC  433

Query  420   CAAAACCCAAA-TTTTCTATGGTTGACCTCGGATCAGGTAGGGATACCCGCTGAACTTAA  478
             ||||||||||| ||||||||||||||||||||||||||||||||||||||||||||||||
Sbjct  434   CAAAACCCAAATTTTTCTATGGTTGACCTCGGATCAGGTAGGGATACCCGCTGAACTTAA  493

Query  479   GCATATCAATAAGCGGAGGAAAAGAAACCAACAGGGATTACCTCAGTAACGGCGAGTGAA  538
             ||||||||||||||||||||||||||||||||||||||||||||||||||||||||||||
Sbjct  494   GCATATCAATAAGCGGAGGAAAAGAAACCAACAGGGATTACCTCAGTAACGGCGAGTGAA  553

Query  539   GCGGTAAAAGCTCAAATTTGAAATCTGGCTCTTTCAGAGTCCGAGTTGTAATTTGTAGAA  598
             |||||||||||||||||||||||||||||||||| ||||||||| |||||||||||||||
Sbjct  554   GCGGTAAAAGCTCAAATTTGAAATCTGGCTCTTTTAGAGTCCGAATTGTAATTTGTAGAA  613

Query  599   GATGCTTCGGGTGTGGTTCCGGTCTAAGTTCCTTGGAACAGGACGTCATAGAGGGTGAGA  658
             ||||||||||||||||||||||||||||||||||||||||||||||||||||||||||||
Sbjct  614   GATGCTTCGGGTGTGGTTCCGGTCTAAGTTCCTTGGAACAGGACGTCATAGAGGGTGAGA  673

Query  659   ATCCCGTATGTGACTGGATACCTATGCTCATGTGAAGCTCTTTCGACGAGTCGAGTTGTT  718
             ||||||||||||||||||||||||||||||||||||||||||||||||||||||||||||
Sbjct  674   ATCCCGTATGTGACTGGATACCTATGCTCATGTGAAGCTCTTTCGACGAGTCGAGTTGTT  733

Query  719   TGGGAATGCAGCTCAAAATGGGTGGTATATTTCATCTAAAGCTAAATATTGGCCAGAGAC  778
             |||||||||||||||||||||| |||||||||| ||||||||||||||||||||||||||
Sbjct  734   TGGGAATGCAGCTCAAAATGGGAGGTATATTTCTTCTAAAGCTAAATATTGGCCAGAGAC  793

Query  779   CGATAGCGCACAAGTAGAGTGATCGAAAGATGAAAAGCACTTTGGAAAGAGAGTTAAACA  838
             ||||||||||||||||||||||||||||||||||||||||||||||||||||||||||||
Sbjct  794   CGATAGCGCACAAGTAGAGTGATCGAAAGATGAAAAGCACTTTGGAAAGAGAGTTAAACA  853

Query  839   GTACGTGAAATTGTTGAAAGGGAAGCGCTTGCAATCAGACTTGCACTTGGTGTTCATCAG  898
             ||||||||||||||||||||||||||||||||||||||||||||||||||||||||||||
Sbjct  854   GTACGTGAAATTGTTGAAAGGGAAGCGCTTGCAATCAGACTTGCACTTGGTGTTCATCAG  913
```

```
Query  899   GGTTTCGTGCCCTGTGTACTTCATCAAGTTCAGGCCAGCATCAGTTTGAGTGGTTAGATA  958
             ||| |||| ||||||||||||||||||||||||||||||||||||||||||||||||||||
Sbjct  914   GGTCTCGTACCCTGTGTACTTCATCAAGTTCAGGCCAGCATCAGTTTGAGTGGTTAGATA  973

Query  959   AAGGCTTGGAGAATGTGGCCCTCTTCGGGGGGTGTTATAGCTCCAGGTGCAATGTAGCCT  1018
             ||||||| |||||||||||||||||||||||||||||||||||| ||||||||||||||||
Sbjct  974   AAGGCTTAGAGAATGTGGCCCTCTTCGGGGGGTGTTATAGCTCTAGGTGCAATGTAGCCT  1033

Query  1019  ACTTGGACTGAGGACCGCGCTTCGGCTAGGATGCTGGCGTAATGGTTGTAAGCGAC  1074
             ||||||||||||||||||||||||||||||||||||||||||||||||||||||||
Sbjct  1034  ACTTGGACTGAGGACCGCGCTTCGGCTAGGATGCTGGCGTAATGGTTGTAAGCGAC  1089


  Database: Nucleotide collection (nt)
    Posted date:  Jan 9, 2023  5:04 PM
  Number of letters in database: 988,652,897,078
  Number of sequences in database:  89,588,680

Lambda      K        H
    1.33    0.621     1.12
Gapped
Lambda      K        H
    1.28    0.460    0.850
Matrix: blastn matrix:1 -2
Gap Penalties: Existence: 0, Extension: 0
Number of Sequences: 89588680
Number of Hits to DB: 0
Number of extensions: 0
Number of successful extensions: 0
Number of sequences better than 0.05: 21040
Number of HSP's better than 0.05 without gapping: 0
Number of HSP's gapped: 26572
Number of HSP's successfully gapped: 26548
Length of query: 1074
Length of database: 988652897078
Length adjustment: 37
Effective length of query: 1037
Effective length of database: 985338115918
Effective search space: 1021795626206966
Effective search space used: 1021795626206966
A: 0
X1: 13 (25.0 bits)
X2: 32 (59.1 bits)
X3: 54 (99.7 bits)
S1: 13 (25.1 bits)
S2: 29 (54.7 bits)
```
